# Interannual variability and trends of combustion aerosol and dust in major continental outflows revealed by MODIS retrievals and CAM5 simulations during 2003–2017

**DOI:** 10.5194/acp-20-139-2020

**Published:** 2020-01-03

**Authors:** Hongbin Yu, Yang Yang, Hailong Wang, Qian Tan, Mian Chin, Robert C. Levy, Lorraine A. Remer, Steven J. Smith, Tianle Yuan, Yingxi Shi

**Affiliations:** 1 Earth Sciences Division, NASA Goddard Space Flight Center, Greenbelt, MD, USA; 2 Pacific Northwest National Laboratory, Richland, WA, USA; 3 Bay Area Environmental Research Institute, Petaluma, CA, USA; 4 NASA Ames Research Center, Moffett Field, CA, USA; 5 Joint Center for Earth Science & Technology, University of Maryland at Baltimore County, Baltimore, MD, USA

## Abstract

Emissions and long-range transport of mineral dust and combustion-related aerosol from burning fossil fuels and biomass vary from year to year, driven by the evolution of the economy and changes in meteorological conditions and environmental regulations. This study offers both satellite and model perspectives on the interannual variability and possible trends of combustion aerosol and dust in major continental outflow regions over the past 15 years (2003–2017). The decade-long record of aerosol optical depth (AOD, denoted as *τ*), separately for combustion aerosol (*τ*_c_) and dust (*τ*_d_), over global oceans is derived from the Collection 6 aerosol products of the Moderate Resolution Imaging Spectroradiometer (MODIS) onboard both Terra and Aqua. These MODIS Aqua datasets, complemented by aerosol source-tagged simulations using the Community Atmospheric Model version 5 (CAM5), are then analyzed to understand the interannual variability and potential trends of *τ*_c_ and *τ*_d_ in the major continental outflows. Both MODIS and CAM5 consistently yield a similar decreasing trend of −0.017 to −0.020 per decade for *τ*_c_ over the North Atlantic Ocean and the Mediterranean Sea that is attributable to reduced emissions from North America and Europe, respectively. On the contrary, both MODIS and CAM5 display an increasing trend of +0.017 to +0.036 per decade for *τ*_c_ over the tropical Indian Ocean, the Bay of Bengal, and the Arabian Sea, which reflects the influence of increased anthropogenic emissions from South Asia and the Middle East in the last 2 decades. Over the northwestern Pacific Ocean, which is often affected by East Asian emissions of pollution and dust, the MODIS retrievals show a decreasing trend of −0.021 per decade for *τ*_c_ and −0.012 per decade for *τ*_d_, which is, however, not reproduced by the CAM5 model. In other outflow regions strongly influenced by biomass burning smoke or dust, both MODIS retrievals and CAM5 simulations show no statistically significant trends; the MODIS-observed interannual variability is usually larger than that of the CAM5 simulation.

## 1 Introduction

Mineral dust resulting from wind erosion in arid and semiarid regions and combustion-related aerosol from burning fossil fuels and biomass are transported at intercontinental and hemispherical scales and deposited into adjacent oceans in large amounts (Yu et al., 2013a; Uno et al., 2009). These aerosols exert important and far-reaching impacts on a variety of aspects of the environment, including air quality and human health (Prospero, 1999; Chin et al., 2007; Anenberg et al., 2014; Tao et al., 2016), the radiation budget (Yu et al., 2006, 2012, 2013b; Song et al., 2018; Yang et al., 2017a, 2018a), cloud lifecycles and precipitation (Kaufman et al., 2005a; Creamean et al., 2013; Wang et al., 2013; Lu et al., 2018), terrestrial and marine ecosystems (Jickells et al., 2005; Yu et al., 2015a), and weather and climate (Yuan et al., 2016; Tao et al., 2018).

Emissions of combustion aerosol and its precursors associated with industrial activities have been changing in response to changes in population, energy structure, and environmental policies. Emissions of particles from biomass burning change with both atmospheric state and human practices. Dust emissions depend strongly on winds and synoptic system meteorology, as well as surface conditions. Meteorological conditions also determine where the particles go and how much is transported across oceans or deposited into oceans. Given large interannual variations in atmospheric circulations and changes in environmental regulations and other drivers on national to regional bases, it is expected that combustion aerosol and dust in the atmosphere may have experienced pronounced year-to-year variation, and significant regional trends may have occurred. Such aerosol changes could have important implications for a variety of environmental issues as mentioned above. Currently it remains a great challenge to quantify the changes in aerosol sources, long-range transport, and environmental impacts.

Satellites are a suitable platform to observe aerosol interannual variability and trends because of their routine sampling over decadal and even multi-decadal timescales with extensive spatial coverage. For example, the Moderate Resolution Imaging Spectroradiometer (MODIS) sensor has been observing the global aerosol system from both the Terra (since February 2000) and Aqua (since July 2002) satellites for more than 15 years (Levy et al., 2018). The MODIS aerosol data record could potentially be extended beyond its lifetime by bridging with retrievals applied to the Visible Infrared Imaging Radiometer Suite (VIIRS) onboard SNPP (launched in late 2011). VIIRS is sufficiently similar in design to MODIS that the MODIS aerosol retrieval algorithms have been adapted for VIIRS inputs, and continuity of the aerosol record is being evaluated (Levy et al., 2015a). Several other sensors have also been detecting aerosols over the globe for at least a decade, including the Advanced Very High Resolution Radiometer (AVHRR) (Zhao et al., 2008), the Total Ozone Mapping Spectrometer (TOMS) (Torres et al., 2002), the Sea-Viewing Wide Field-of-view Sensor (SeaWiFS) (Hsu et al., 2012), the Multi-angle Imaging SpectroRadiometer (MISR) (Kahn et al., 2007), the Ozone Monitoring Instrument (OMI) (Torres et al., 2007), the Cloud–Aerosol Lidar with Orthogonal Polarization (CALIOP) (Winker et al., 2013), and the Infrared Atmospheric Sounding Interferometer (IASI) (Capelle et al., 2018). These instruments have been acquiring long-term datasets of aerosol optical depth (AOD) and several particle properties (e.g., size, shape, and absorption). CALIOP also provides critical information for the aerosol vertical distribution. These data records provide an opportunity to characterize aerosol interannual variability and trends over a decadal timescale, although various uncertainties in aerosol retrievals and sensor degradation could complicate trend detection (Li et al., 2009).

In the last decade, a number of studies have analyzed satellite data to detect aerosol interannual variability and trends on regional or global scales, mostly focusing on the total aerosol without distinguishing aerosol types from the data (Mishchenko et al., 2007; Zhao et al., 2008; Zhang and Reid, 2010; Zhang et al., 2017; Hsu et al., 2012; Chin et al., 2014; Yu et al., 2015b; Jongeward et al., 2016; Alfaro-Contreras et al., 2017; de Leeuw et al., 2018; Chedin et al., 2018). Determining the types of aerosol responsible for the detected AOD variability or trends remains elusive. A distinction between largely natural dust and largely man-made combustion aerosol should provide greater insight into changes in aerosol sources and long-range transport than does total aerosol. Dust and combustion aerosol are also different in their many properties and a variety of environmental impacts.

The overall objective of this study is to characterize the interannual variability and possible trends of combustion aerosol and dust, separately, in major continental outflow regions over the last 2 decades. Specific science questions we address include the following: how has industrial pollution changed on a regional basis in recent decades? How do episodic dust and biomass burning smoke vary from year to year? Are there regional trends over the past 15 years? Why does the aerosol change? Is the AOD trend consistent with that of aerosol emissions? We proceed to achieve the objective through a combined analysis of satellite retrievals from the MODIS instrument and model simulations from the Community Atmosphere Model version 5 (CAM5). In contrast to previous work, this study examines combustion aerosol and dust separately from the MODIS retrievals. We also use the CAM5 simulations with aerosol source-tagging capability to do source attribution and chemical characterization for the MODIS-detected combustion AOD variability and trends in major outflow regions of continental aerosol. We focus on the outflow regions over water bodies because the MODIS retrievals of AOD and particle properties are more accurate over ocean than over land, and thus we can better separate combustion aerosol from dust.

The rest of paper is organized as follows. Section 2 gives a brief description of the MODIS aerosol products and the CAM5 model setups. In Sect. 3, we first describe the derivation of dust and combustion aerosol components specific to the MODIS Collection 6 (C6) data (3.1), which is an important update to previous studies based on the earlier MODIS data collections (Kaufman et al., 2005b; Yu et al., 2009). This is followed by the seasonal and spatial characteristics of dust and combustion AOD climatology based on both MODIS retrievals and CAM5 simulations (3.2). Then we analyze the interannual variability and possible trends of *τ*_c_ and *τ*_d_ in major combustion aerosol and dust outflows from the MODIS Aqua retrievals complemented by the CAM5 simulations (3.3). Section 4 summarizes major conclusions from the combined MODIS and CAM5 analysis and discusses major issues of relevance.

## 2 Description of MODIS retrievals and CAM5 simulations of aerosols

### 2.1 MODIS aerosol retrievals

The twin MODIS sensors have been flying on Terra (with equatorial overpassing time at about 10:30 local time) since February 2000 and on Aqua (with equatorial overpassing time at about 13:30 local time) since June 2002. The MODIS instrument is a 36-channel radiometer covering wavelengths from deep blue (0.41 μm) to the thermal infrared. The instrument samples a broad swath of about 2330 km at a spatial resolution of 500 m or 1 km (depending on channel) at nadir. As such the MODIS sensors have been acquiring essential data for atmospheric and surface properties with nearly daily global coverage for a period of more than 15 years. Three complementary aerosol retrieval algorithms: namely, the Dark Target (DT), the Deep Blue (DB), and the Multi-Angle Implementation of Atmospheric Correction (MAIAC) algorithms have been developed, operated, maintained, and updated at NASA Goddard Space Flight Center. The consistent retrieval algorithms have been applied to both MODIS sensors during their lifetime. In this study, we use aerosol retrievals from the DT algorithm that was developed to retrieve aerosol loading and properties over dark surfaces, including ocean–water (Remer et al., 2005) and vegetated land (Levy et al., 2007). Because of its wide spectral range and the simplicity of the dark ocean surface, the DT algorithm has the capability of retrieving AOD with high accuracy and information on particle size (in the form of the Ångström exponent, effective radius, or fine-mode fraction – FMF) (Remer et al., 2005; Levy et al., 2013).

In this study, we use the MODIS DT C6 over-ocean aerosol data records from both Terra and Aqua, i.e., the Level 3 daily product gridded to 1° × 1° (i.e., MOD08D3 and MYD08D3, respectively) (Levy and Hsu, 2015b, c). In previous studies, we developed an approach of distinguishing dust from combustion aerosol by using MODIS retrievals of total AOD (*τ*) and FMF (*f*) (Kaufman et al., 2005b; Yu et al., 2009). Both *τ* and *f* refer to properties at 550 nm hereafter, except if specified otherwise. In this approach, *τ* and fine-mode AOD (*f τ*) are assumed to be composed of marine aerosol, dust, and combustion aerosol, i.e.,

(1) $$\tau =\tau_{\text{m}}+\tau_{\text{d}}+\tau_{\text{c}},$$

(2) $$f\tau =f_{\text{m}}\tau_{\text{m}}+f_{\text{d}}\tau_{\text{d}}+f_{\text{c}}\tau_{\text{c}},$$

where the subscripts m, d, and c refer to marine aerosol, dust, and combustion aerosol, respectively. The marine aerosol refers collectively to the primary and secondary particles associated with wave-breaking sea spray, including sea salt, marine organics, and sulfate produced from dimethyl sulfide (DMS). The combustion aerosol, also referred to as “pollution” or “anthropogenic” aerosol in previous studies (Kaufman et al., 2005b; Yu et al., 2008, 2009), results mainly from the burning of fossil fuels and biomass. It also includes contributions from volcanic activities. Based on Eqs. (1) and (2), *τ*_d_ and *τ*_c_ can be calculated from the MODIS-retrieved *τ* and *f*, with appropriate parameterizations for *f*_d_, *f*_c_, *f*_m_, and *τ*_m_ (see details in Kaufman et al., 2005b; Yu et al., 2009). In earlier versions (e.g., C4 and C5), while *τ*_m_ was parameterized as a function of wind speed, *f*_d_, *f*_c_, and *f*_m_ were determined from retrieved *f* in selected regions and seasons for which a specific aerosol type dominates (Kaufman et al., 2005b; Yu et al., 2009). Given that the MODIS retrievals, in particular *f*, are sensitive to many details of the retrieval algorithm and instrument calibration, the characteristic *f*_d_, *f*_c_, and *f*_m_ should be considered “dynamical”. As discussed in Levy et al. (2013, 2018), several aspects of the MODIS DT retrieval algorithm and the instrument calibration have evolved from C5 to C6, which warrants a reassessment of the dust–combustion separation approach to ensure the consistent use of the MODIS C6 product without possibly introducing additional errors (Yu et al., 2009). This is addressed in Sect. 3.1.

### 2.2 CAM5 simulations

To investigate interannual variability and trends of global aerosols, a 37-year simulation covering 1979–2015 has been carried out with time-varying emissions of aerosol and precursors using the Community Atmosphere Model version 5.3 (CAM5–MAM3). In the three-mode modal aerosol module (MAM3; Liu et al., 2012), aerosol species, including sulfate (SO_4_), black carbon (BC), primary organic matter (POM), second organic aerosol (SOA), mineral dust, and sea salt, are predicted with improved convective transport and wet scavenging schemes (Wang et al., 2013). At 1.9° latitude by 2.5° longitude horizontal grids and 30 vertical layers, model wind fields are nudged to the NASA Modern Era Retrospective Analysis for Research and Applications (MERRA) reanalysis (Rienecker et al., 2011) every 6 h. This long-term historical simulation ended in 2015 because of the lack of availability of the MERRA reanalysis. Monthly anthropogenic (version 20160726, 1979–2014) and open biomass burning (version 20161213, 1979–2015) emissions are taken from the Coupled Model Intercomparison Project Phase 6 (CMIP6) emission database (Hoesly et al., 2018; van Marle et al., 2017). For anthropogenic emissions in the year 2015, emission data are interpolated from the SSP2–4.5 (Shared Socioeconomic Pathways 2–4.5) forcing scenarios (Riahi et al., 2017). Note that the term biomass burning in this work refers to open burning, such as forest fires and agricultural waste burning on fields. Biofuel combustion is categorized as anthropogenic emissions.

**Figure 1.**
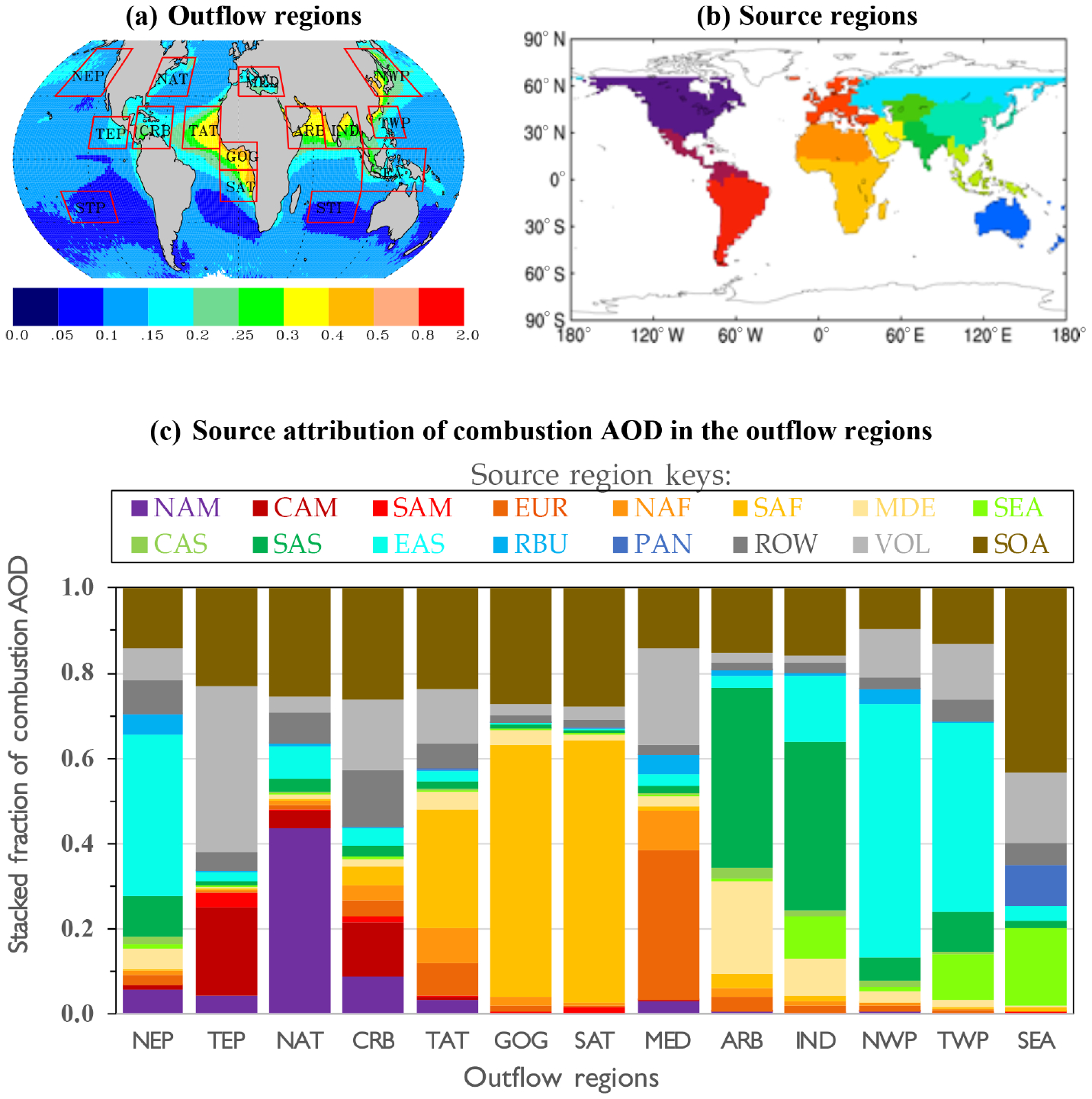
Illustration of (**a**) 13 continental outflow regions (covering only the water-body portion of boxes) plus two remote regions (STI and STP) overlaid onto a map of MODIS annual mean AOD climatology (color bar), (**b**) 14 source regions used in the CAM5 tagged simulations, and (**c**) CAM5 source attributions (i.e., stacked fractions from 14 source regions plus VOL and SOA types) of combustion AOD in the outflow regions based on 2003–2015 simulations.

To understand the source attribution and chemical components of combustion aerosol in each of 13 continental outflow regions (Fig. 1a) in this study, we utilize the CAM5 aerosol source-tagging capability (Wang et al., 2014; Yang et al., 2017b, c, 2018a, b). With this capability, BC, POM, and sulfate (and precursor gases SO_2_ and DMS) from a variety of sources are explicitly tagged and tracked in the model. This tagging technique can offer insights into contributions by different regions and sources, which satellite retrievals cannot provide. For this study, aerosols and their precursor emissions, including SO_2_, sulfate, BC, and POM, are tagged with respect to 14 source regions (Fig. 1b) as defined in the Hemispheric Transport of Air Pollution Phase 2 (HTAP2), namely North America (NAM), Central America (CAM), South America (SAM), Europe (EUR), North Africa (NAF), southern Africa (SAF), the Middle East (MDE), Southeast Asia (SEA), Central Asia (CAS), South Asia (SAS), East Asia (EAS), Russia–Belarus–Ukraine (RBU, hereafter Russia), Pacific–Australia–New Zealand (PAN, hereafter the southern Pacific), and rest of the world (ROW). The tagging also applies to two specific sources for sulfate: volcanic emissions (VOL) and DMS chemistry (DMS). Note that secondary organic aerosol (SOA) is not included in the tagging in this study, although this is calculated by the model. Figure 1c shows the linkage between the continental outflow regions and source regions as reflected by the 2003–2015 climatology of CAM5 source attributions of combustion AOD in each of the outflow regions.

**Figure 2.**
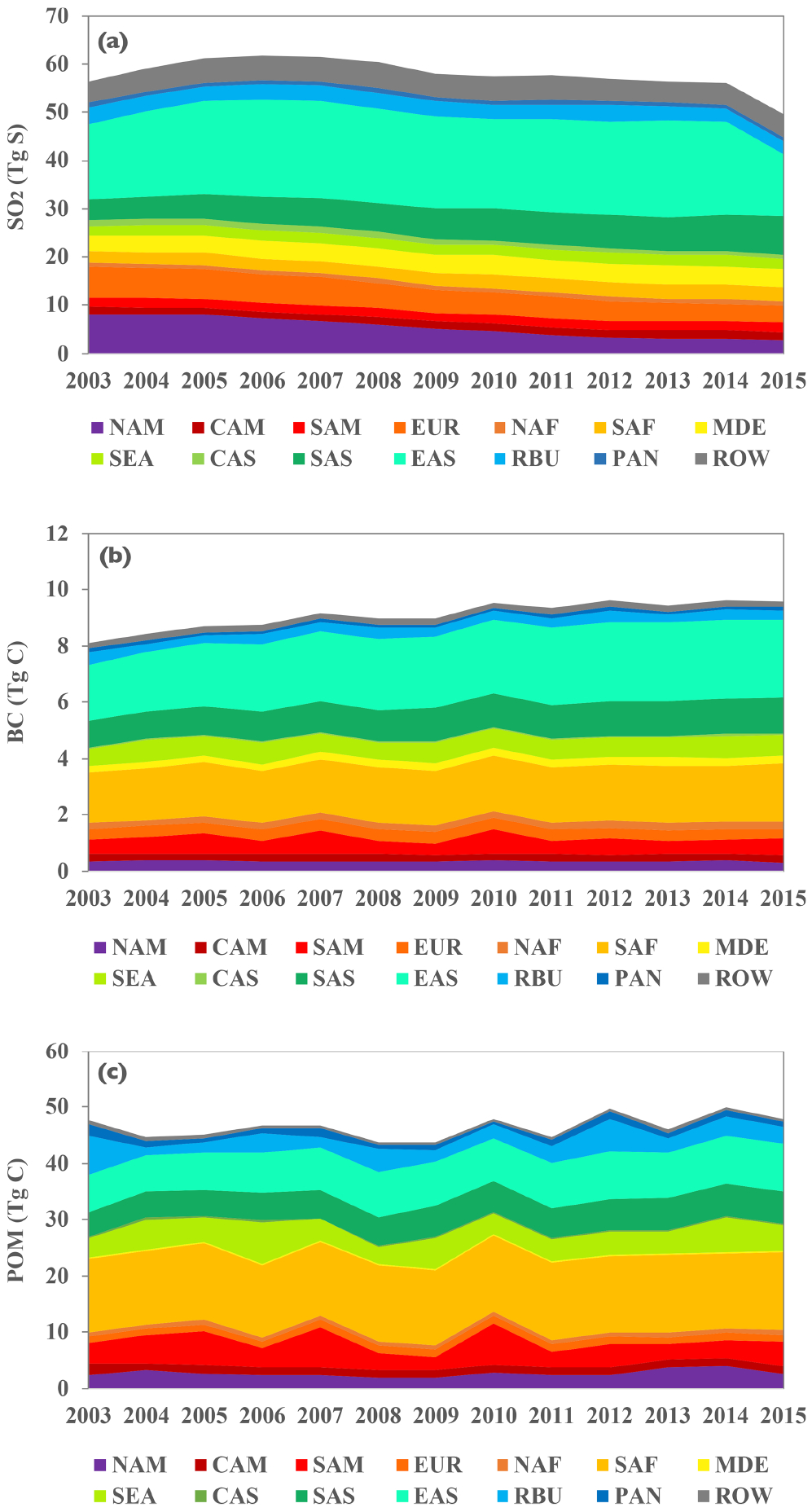
The 2003–2015 source-region stacked global annual emissions of SO_2_ (**a**), BC (**b**), and POM (**c**) used in the CAM5 model.

Figure 2 displays the global annual anthropogenic and biomass burning emissions of SO_2_, BC, and POM from 2003 to 2015 that are stacked from the 14 source regions. There are several prominent changes in regional emissions. The SO_2_ emissions from North America and Europe have been consistently decreasing from 2003 to 2015, with an accumulated reduction of 67 % and 45 % of their 2003 levels, respectively. In contrast, the SO_2_ emissions from South Asia have continuously increased by 80 % over this time period. The SO_2_ emissions from East Asia have taken a more complicated trajectory, increasing from 2003 until 2007 and then fluctuating from year to year until a sharp decline occurred in 2015. Overall, the East Asian SO_2_ emissions have decreased by 16 % from 2003 to 2015. The different trajectories between East Asian and South Asian SO_2_ emissions are generally consistent with satellite observations (Li et al., 2017). East Asia and southern Africa are the two largest emitters of BC and POM, with the former dominated by anthropogenic activities and the latter by biomass burning. In contrast to the region’s SO_2_ emissions discussed earlier, BC and POM emissions in East Asia increased continuously by 40 % and 27 %, respectively, in this dataset. For both BC and POM emissions, large interannual variability is shown in major biomass burning regions, such as South America, southern Africa, Southeast Asia, and Russia.

## 3 Results

### 3.1 Characteristic FMFs for distinct aerosol types from MODIS C6 products

**Figure 3.**
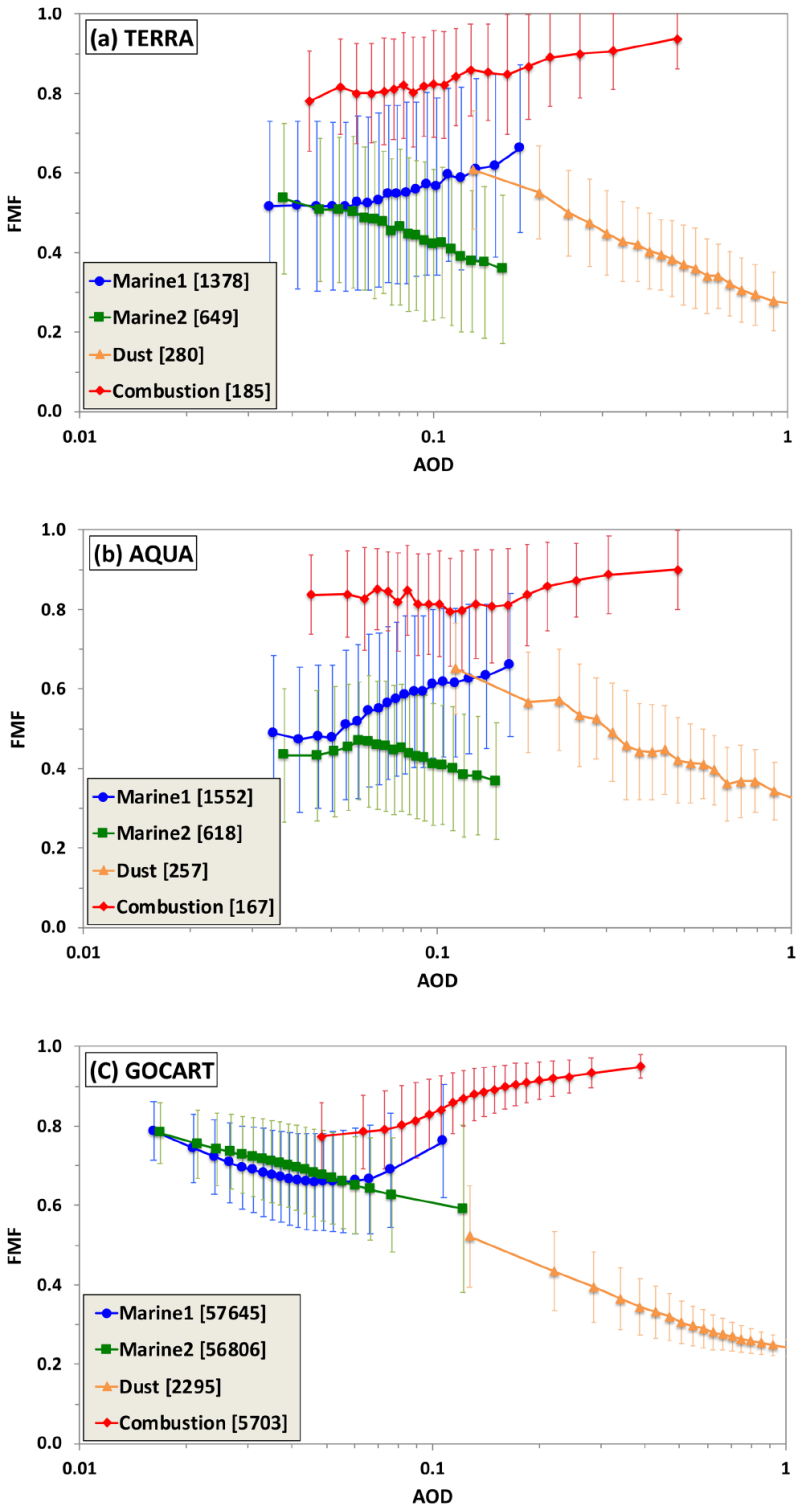
Aerosol fine-mode fraction (FMF) as a function of AOD in three regions representative of marine aerosol, dust, and combustion aerosol derived from the MODIS observations on Terra (**a**) and Aqua (**b**) and the GOCART model (**c**). Marine aerosol regime is categorized into Marine1 and Marine2, representing May–October and November–April, respectively. All the data are sorted into 20 AOD bins with equal data points (numbers in brackets), and the mean (marked with symbol) and standard deviation (vertical bar) of FMF are calculated.

As discussed in Sect. 2.1, a caveat for the MODIS-based dust–combustion separation algorithm is that *f*_d_, *f*_c_, and *f*_m_ derived from the MODIS Terra C4 (Kaufman et al., 2005b) and C5 (Yu et al., 2009) products should not be used directly for the MODIS C6 on Terra and Aqua. To maintain a self-consistent use of the MODIS C6 products, here we first reassess the characteristic FMF for individual aerosol types (i.e., *f*_d_, *f*_c_, *f*_m_) and then re-derive combustion and dust AOD by following the method in previous studies (Kaufman et al., 2005b; Yu et al., 2009). Figure 3 shows how the MODIS-retrieved FMF changes with AOD, separately for Terra (a) and Aqua (b), in three regions respectively dominated by outflows of combustion aerosol (North Atlantic Ocean, in summer), dust (eastern North Atlantic Ocean just off the coast of North Africa, in summer), and marine aerosol (southern Indian Ocean, full year). (These regions are defined the same as in previous studies to facilitate comparison.) The data in the southern Indian Ocean are stratified into two time periods when the region can be somewhat influenced by transported smoke in one time period (May–October, denoted as “Marine1”) and by transported dust in the other (November–April, denoted as “Marine2”). For comparison, similar FMF–AOD relationships (Fig. 3c) are derived from the Goddard Chemistry Aerosol Radiation Transport (GOCART) model. In the GOCART model, sulfate, POM, and BC are counted as fine-mode aerosol, whereas dust and sea-salt include both fine-mode and coarse-mode with a division at a radius of 1 μm. Generally, MODIS retrievals and GOCART simulations consistently show similar FMF changes with respect to AOD. In the combustion-dominated outflow region, FMF increases with increasing AOD, which is consistent with the addition of fine-mode combustion particles into the relatively coarser (smaller FMF) background marine aerosol. In the dust outflow regions, on the contrary, FMF decreases with increasing AOD, which is consistent with the addition of coarser dust particles (hence smaller *f*) on the background marine aerosol. For the marine-aerosol-dominated southern Indian Ocean, FMF remains relatively constant, with increasing AOD for AOD up to about 0.06, for both the Marine1 and Marine2 clusters. Then FMF increases (decreases) with increasing AOD for the Marine1 (Marine2) cluster. This bifurcating pattern reflects the influence of long-range-transported smoke and dust in the Marine1 and Marine2 cluster, respectively.

We derive from Fig. 3 the characteristic *f*_c_, *f*_d_, and *f*_m_ for individual aerosol types as follows. On one hand, *f*_c_ for combustion aerosol and *f*_d_ for dust are taken as the average *f* for the largest AOD bin in the combustion-aerosol- and dust-dominated region, respectively. In the largest AOD bin, a contribution from the marine background is at a minimum and is neglected. On the other hand, *f*_m_ for marine aerosol is the average *f* in the smallest AOD bin that represents the marine background. Table 1 lists the characteristic *f*_c_, *f*_d_, and *f*_m_ derived from the MODIS C6 data (both Terra and Aqua) and the GOCART model. The three datasets consistently show that *f*_c_ is substantially larger than *f*_d_, with *f*_m_ in between. However, some notable differences do exist among them. The MODIS-based characteristic *f*_c_, *f*_d_, and *f*_m_ differ by 0.03, 0.05, and 0.07, respectively, between Terra and Aqua. These differences may have resulted from differences in instrument calibrations (Levy et al., 2018). The major difference between GOCART and MODIS exists for *f*_m_, with the GOCART *f*_m_ of 0.78 being much higher than the MODIS values of 0.55 for Terra and 0.48 for Aqua.

The characteristic *f*_c_, *f*_d_, and *f*_m_ derived from the MODIS Terra C6 products in this study are also compared with those previously derived from the C4 (Kaufman et al., 2005b) and C5 (Yu et al., 2009), as shown in Table 2. Although *f*_c_ remains nearly unchanged, notable but opposite changes have occurred for *f*_d_ and *f*_m_ in the course of evolving data collections. On one hand, *f*_d_ has decreased from 0.51 (C4) and 0.37 (C5) to 0.26 (C6). On the other hand, *f*_m_ has increased from 0.32 (C4) and 0.45 (C5) to 0.55 (C6). These changes highlight the importance of the reassessment above to ensure a consistent use of the MODIS products. Applying the characteristic FMF values derived from the earlier versions of data (C5 or C4) to the MODIS C6 products would introduce large uncertainty to the calculation of *τ*_d_ and *τ*_c_. Similarly, caution should be exercised when applying the MODIS-based characteristic FMF values to the VIIRS over-ocean aerosol products, despite their similarity in instrument design and the Dark Target aerosol retrieval algorithm (Levy et al., 2015a). It is strongly recommended that a similar assessment of the characteristic FMFs be carried out with the VIIRS retrievals.

### 3.2 Climatology of combustion aerosol and dust from MODIS and CAM5

**Figure 4.**
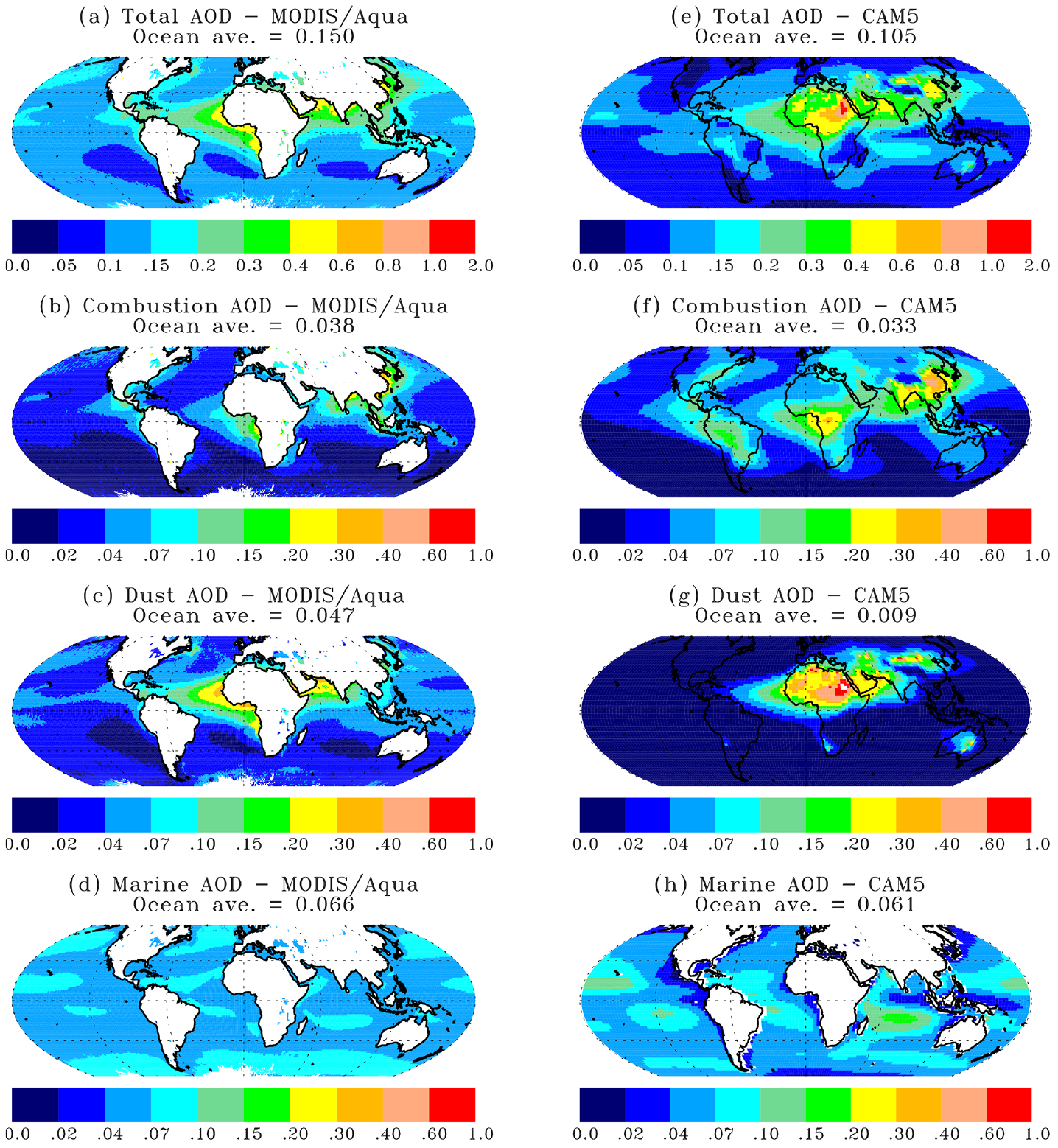
The 2003–2015 AOD climatology (**a, e**) and its partition into combustion, dust, and marine aerosol as derived from MODIS Aqua observations (**a–d**) and CAM5 simulations (**e–h**). Note that the color scale for total AOD (**a, e**) is different from that of its components.

We apply the C6-based characteristic FMF values to derive *τ*_c_ and *τ*_d_ from the MODIS Terra and MODIS Aqua C6 products by following a similar method as described in Yu et al. (2009). *τ*_m_ is parameterized as a function of daily 10 m wind speed from the Modern Era Retrospective Analysis for Research and Applications version 2 (MERRA-2) (Geralo et al., 2017) following the average of two relationships from Kiliyanpilakkil and Meskhidze (2011) and Mulcahy et al. (2008). Figure 4 shows the 2003–2015 MODIS Aqua climatology of annual mean total AOD and its partition into *τ*_c_, *τ*_d_, and *τ*_m_ over ocean (left panels).

The figure shows that the spatial patterns of combustion and dust AOD over ocean derived from the MODIS retrievals are consistent with their largely well-known upwind continental sources. Major outflow regions with elevated combustion AOD include the following: off the coast of the Asian continent over the North Pacific, off the coast of the eastern US over the North Atlantic, off the coast of the Indian subcontinent over the tropical Indian Ocean and the Bay of Bengal, in the Gulf of Mexico and eastern tropical Pacific Ocean, and over the tropical Atlantic Ocean downwind of the African biomass burning region. Major dust outflow regions include the tropical Atlantic Ocean and the Caribbean Basin, the North Pacific Ocean, and the Arabian Sea–tropical Indian Ocean. Although dust has been observed over the southeastern Atlantic Ocean (Vickery et al., 2013; Formenti et al., 2019), the MODIS-derived dust AOD is likely overestimated. The year-round presence of stratocumulus clouds in the region poses a major challenge for aerosol retrievals, probably yielding a high AOD bias, low FMF bias, and hence a high bias of dust AOD. In fact, the MODIS-derived *τ*_c_ is higher than that derived from MISR and IASI south to the Equator (Yu et al., 2019). Over the tropical Pacific Ocean, a long belt of somewhat elevated dust AOD may also indicate an artifact due to possible cloud contamination. The seasonal variations of *τ*_c_ and *τ*_d_ are shown in Fig. S1 of the Supplement.

The CAM5 simulations of *τ* and its components (*τ*_c_, *τ*_d_, and *τ*_m_) over both land and ocean are shown in the right panels of Fig. 4. Here for the purpose of comparison with the MODIS data, we derive the CAM5 marine AOD as a sum of the AOD of sea salt and DMS-generated SO_4_ and the combustion AOD as a sum of the AOD of SO_4_ (excluding those generated from DMS chemistry), BC, POM, and SOA. The CAM5 model captures the major outflows of combustion aerosol well. On global ocean average, the CAM5 *τ*_c_ (0.033) is lower than the MODIS retrieval of 0.038 by about 13 %. However, the CAM5 model substantially underestimates the dust outflows into the North Pacific Ocean and the tropical Atlantic Ocean, although it reproduces the MODIS-derived dust outflow into the Arabian Sea and the tropical Indian Ocean. On the basis of the global ocean average, the MODIS-derived *τ*_d_ of 0.047 is about a factor of 5 higher than the 0.009 from the CAM5 model. The MODIS-derived global mean *τ*_m_ is 0.066 at 550 nm, which agrees well with the climatology based on AERONET observations (Kaufman et al., 2001; Smirnov et al., 2002). Although the CAM5 model agrees with the global mean of MODIS-derived marine AOD within 8 %, the model is higher at middle to high latitudes but lower in tropical regions than the MODIS-derived marine AOD. In summary, substantial disparity in dust AOD exists between the MODIS retrieval and CAM5 simulations. Although the cloud contamination issues discussed earlier may have biased the MODIS-derived dust AOD higher, this alone is unlikely to adequately explain the factor of 5 difference between MODIS retrievals and CAM5 simulations. We will further discuss this issue by applying insight gained from a regional analysis.

**Figure 5.**
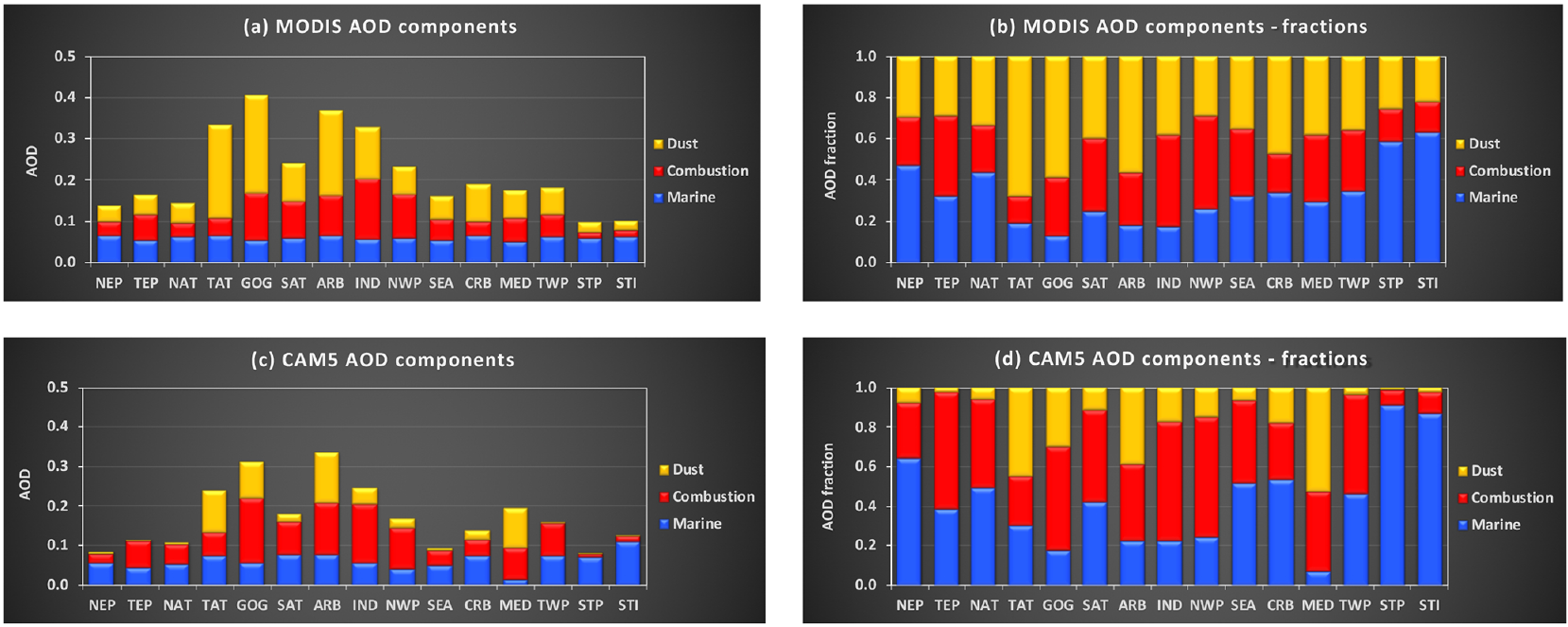
A climatology of aerosol components (i.e., marine, combustion, and dust) in 15 selected regions (see Fig. 1a for definition) based on the MODIS Aqua 2003–2017 observations for (**a**) AOD and the (**b**) stacked AOD fraction as well as the CAM5 2003–2015 simulations for (**c**) AOD and the (**d**) stacked AOD fraction. The contribution by marine, combustion, and dust aerosol is marked in blue, red, and yellow, respectively. The sum of the fractional contribution by the three components amounts to 1 in (**b**) and (**d**)

The partitioning of AOD is further examined on a regional basis, focusing on 13 major continental outflow regions and two pristine regions as illustrated in Fig. 1a. Figure 5 shows the regional partition of total AOD into marine aerosol, combustion aerosol, and dust on an annual mean basis from both MODIS Aqua retrievals and CAM5 simulations. Both the absolute value and fractional contribution of AOD by the three components are shown in stacked bar charts with different colors. The sum of the fractional contributions by the three components amounts to 1.0 in the stacked bar charts. The seasonal variability of regional aerosol components based on the MODIS retrievals is shown in Fig. S2 in the Supplement. The MODIS retrievals show that marine AOD falls into a range of 0.053–0.067 across all the regions. In the pristine regions (STP and STI), *τ*_m_ accounts for about 60 % of total AOD. The remainder is partitioned into *τ*_c_ of 0.015 and *τ*_d_ of 0.024 on average. For comparison, the CAM5 simulations suggest that marine AOD accounts for about 90 % of total AOD in these pristine regions, with the remaining 10 % contributed largely by combustion aerosol. Although the MODIS-retrieved *τ*_c_ and *τ*_d_ levels in these pristine regions do represent the smallest continental influences among all the regions, they may be subject to uncertainty or bias. For example, the MODIS aerosol retrievals are inevitably contaminated by imperfect cloud screening, possibly resulting in a high AOD bias and low FMF bias. The parameterization of *τ*_m_ could also introduce uncertainty to the derived *τ*_c_ and *τ*_d_. Both the issues would affect *τ*_d_ more than *τ*_c_. It is, however, formidable to rigorously assess the bias without acquiring independent measurements of dust optical depth with high accuracy. Nevertheless, if we assume that these remote regions are perfectly pristine without any influence from combustion aerosol and dust, then the 0.015 and 0.024 could represent an upper bound of potential bias associated with the MODIS-derived *τ*_c_ and *τ*_d_, respectively. If it is further assumed that a similar magnitude of bias exists over the global ocean, this yields a bracket for the MODIS-based global ocean average of 0.023–0.038 for *τ*_c_ and 0.023–0.047 for *τ*_d_. For comparison, *τ*_c_ derived from the earlier versions of the MODIS product is 0.033 (Kaufman et al., 2005c) and 0.035 (Yu et al., 2009) for C4 and C5, respectively. The CAM5 *τ*_c_ of 0.033 also falls into the range of the adjusted MODIS retrievals. On the other hand, the *τ*_d_ of 0.009 from the CAM5 model still accounts for no more than 38 % of the MODIS retrieval. This may suggest that a great deal of effort is needed to improve the model simulations of dust.

In comparison to the remote regions, the percentage of *τ*_m_ contribution from the MODIS retrieval is decreased to 13 %–47 % in the major outflow regions, with the magnitude depending on the influence of continental aerosol sources. The *τ*_m_ contribution is more than 40 % in the northeastern Pacific Ocean (NEP) and the northern Atlantic Ocean (NAT). The *τ*_m_ contribution drops below 20 % in the Gulf of Guinea (GOG), the Arabian Sea (ARB), northern Indian Ocean and the Bay of Bengal (IND), and the tropical Atlantic Ocean (TAT) because of strong influences of desert dust and combustion-related aerosol from the upwind continent. The MODIS retrievals in Fig. 5 further indicate relative contributions of *τ*_c_ and *τ*_d_ in the outflow regions. The three most dust-dominated regions are TAT, GOG, and ARB, where *τ*_d_ *>* 0.2 and accounts for more than 50 % of *τ*. The three highest regional *τ*_c_ (*>*0.1) values occur in IND, GOG, and the northwestern Pacific Ocean (NWP), which respectively account for about 44 %, 28 %, and 45 % of *τ*. The lower percentage of *τ*_c_ in GOG results from the coexistence of high *τ*_d_ of 0.237 in the region. Several regions have a lower *τ*_c_ that, however, accounts for a higher percentage of *τ* due to small share of dust, including the tropical eastern Pacific (TEP), the southeastern Atlantic Ocean (SAT), the Mediterranean Sea (MED), the southeastern Asia (SEA), and the tropical western Pacific Ocean (TWP). As discussed earlier, the contribution of *τ*_c_ in SAT may have been underestimated because the persistence of cloudiness in the region has likely resulted in the high bias to *τ* and *τ*_d_. In comparison to the MODIS retrievals, the CAM5 simulations generally yield much smaller dust fractions. One exception is the MED region where the CAM5 dust fraction is the largest among all the regions.

**Figure 6.**
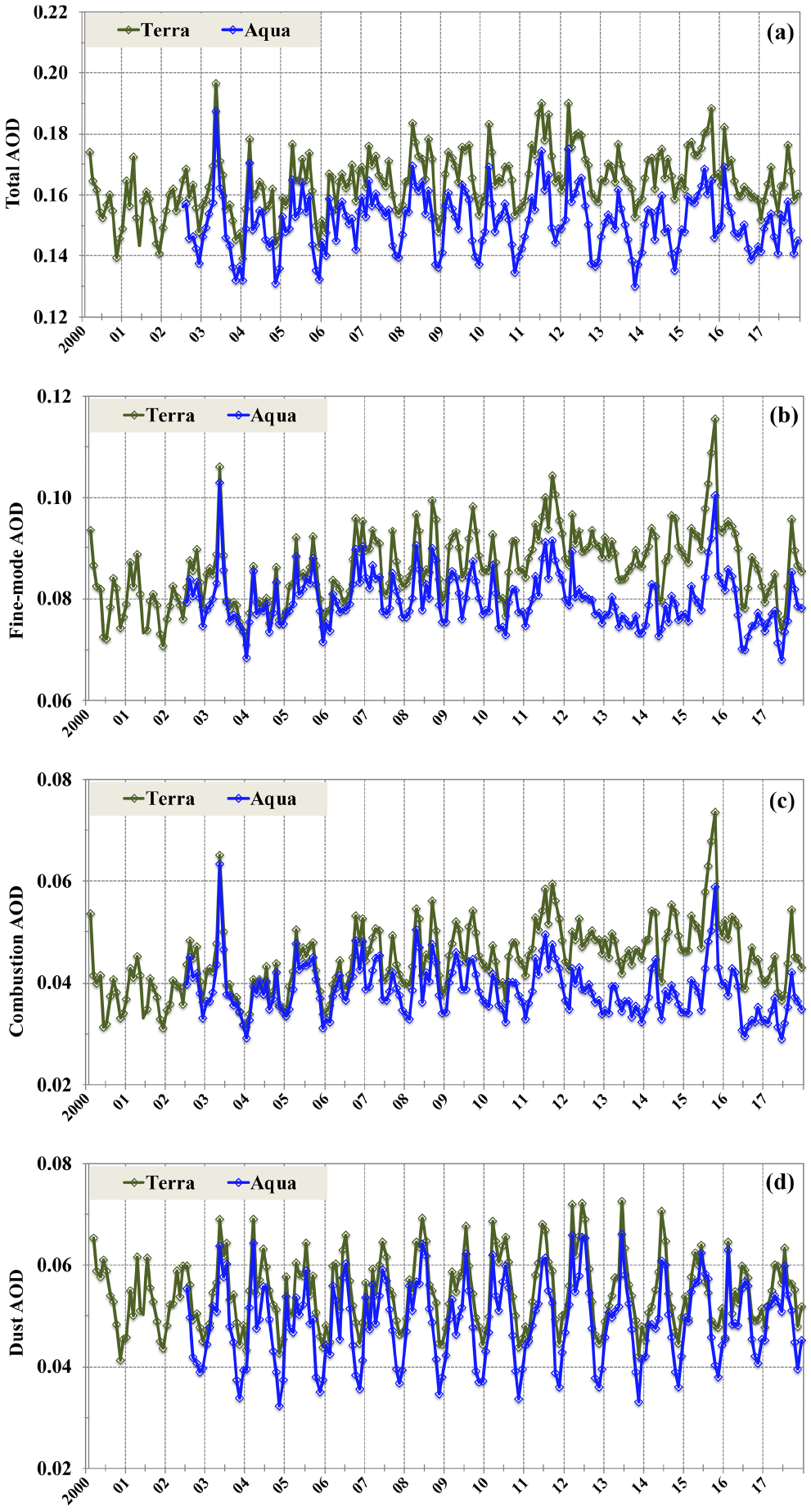
Comparison of global ocean, monthly average total AOD (**a**), fine-mode AOD (**b**), combustion AOD (**c**), and dust AOD (**d**) derived from the MODIS Terra (green) and MODIS Aqua (blue) observations over the 2000–2017 period.

**Figure 7.**
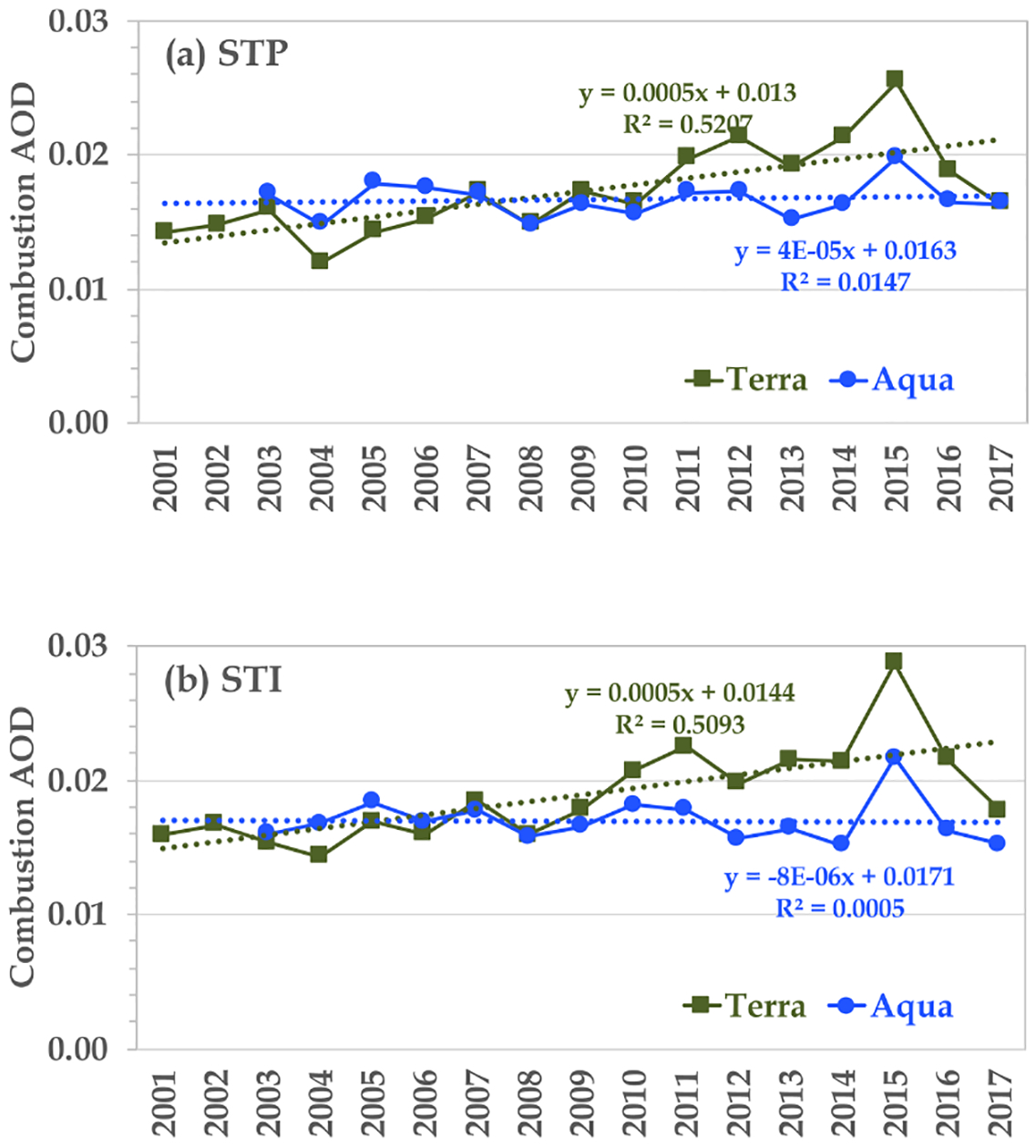
MODIS retrievals of *τ*_c_ in (**a**) the southern tropical Pacific Ocean (STP) and (**b**) the southern tropical Indian Ocean (STI). While Aqua retrievals show no trend, Terra retrievals indicate a statistically significant (*p<*0.001) and increasing *τ*_c_ trend of 0.005 per decade.

Although the MODIS Terra retrievals (not shown) bear great resemblance to the patterns revealed by the MODIS Aqua retrievals (Fig. 4), the two differ somewhat in the magnitude of AOD. Previous studies have found an increasing trend of total AOD over the global ocean from analyzing the MODIS Terra retrievals but no trend from the MODIS Aqua retrievals (Zhang et al., 2017; Levy et al., 2018). It has been argued that this MODIS Terra trend of global ocean mean AOD is spurious, which could have resulted from imperfect calibrations being implemented to correct radiances observed by the aging Terra platform or different sampling due to changing clouds and viewing angles from late morning to early afternoon, among other issues (Levy et al., 2018). Here we further explore this issue by examining if this spurious trend affects combustion and dust AOD differently. Figure 6 compares the time series of MODIS Terra and MODIS Aqua monthly retrievals averaged over global oceans (60° S–60° N) for total, fine-mode, combustion, and dust AOD. Although the twin MODIS sensors observe the consistent month-to-month variations for both total and component AODs, clear offsets exist between Terra and Aqua datasets, with the MODIS Terra values being higher than the MODIS Aqua values in general. The offsets have also become larger since 2007 (particularly after 2012) for total, fine-mode, and combustion AOD. Furthermore, an increasing trend is clearly shown in the MODIS Terra retrievals of total, fine-mode, and combustion AOD but is not evident in the MODIS Aqua retrievals. Although the dust AOD, in particular its minimum value of annual cycle, is lower in Aqua than in Terra, there is no clear trend in both MODIS Terra and MODIS Aqua retrievals. The increasing trends in the MODIS Terra retrievals but lack of any in the MODIS Aqua retrievals do not represent realistic late morning and early afternoon differences in aerosol, in particular on the basis of the global ocean average (Levy et al., 2018). As a sanity check, we further examine AODs in the two remote regions, i.e., STP and STI (as defined in Fig. 1a), where the influence by continental outflows of aerosol is minimal (as clearly shown in Figs. 4 and 5). A similar strategy has been applied in the literature (e.g., Zhang and Reid, 2010; Zhang et al., 2017), and Alfaro-Contreras et al. (2017) adjusted the MODIS Terra AOD trends by subtracting the detected trend in the remote ocean regions. As shown in Fig. 7, the MODIS Aqua retrievals are consistent with the anticipation that the combustion AOD in such relatively pristine regions has had no consistent trend over the last 2 decades. In contrast, the MODIS Terra retrievals of *τ*_c_ show an increasing trend of +0.005 per decade (*p<*0.001) in both regions. For comparison, Zhang et al. (2017) detected an increasing trend of +0.006 per decade for total AOD in the remote ocean regions. Based on the above analysis, we will only use the MODIS Aqua retrievals to examine interannual variability and possible trends of combustion and dust AOD on a regional basis.

### 3.3 Interannual variability and trends of combustion aerosol and dust in major continental outflows

In this section, we use the MODIS Aqua retrievals over the last 15 years (2003–2017) to study regional AOD interannual variability and trends, separately for combustion aerosol and dust, focusing on the 13 major continental outflow regions defined in Fig. 1a. The significance of a trend is checked with the Student’s *t* test at two levels (*p<*0.01 or *p<*0.05). The MODIS retrievals are compared with the CAM5 simulations over the 2003–2015 period. The CAM5 source-tagging simulations are used to facilitate the interpretation of the MODIS-observed *τ*_c_ changes in terms of major source regions and chemical composition. Although the same analysis has been performed for both *τ*_c_ and *τ*_d_ in all the outflow regions, we only discuss results with salient features in the following and place the remaining results in the Supplement.

#### 3.3.1 Declining trends of *τ*_c_ in North American and European outflows

**Figure 8.**
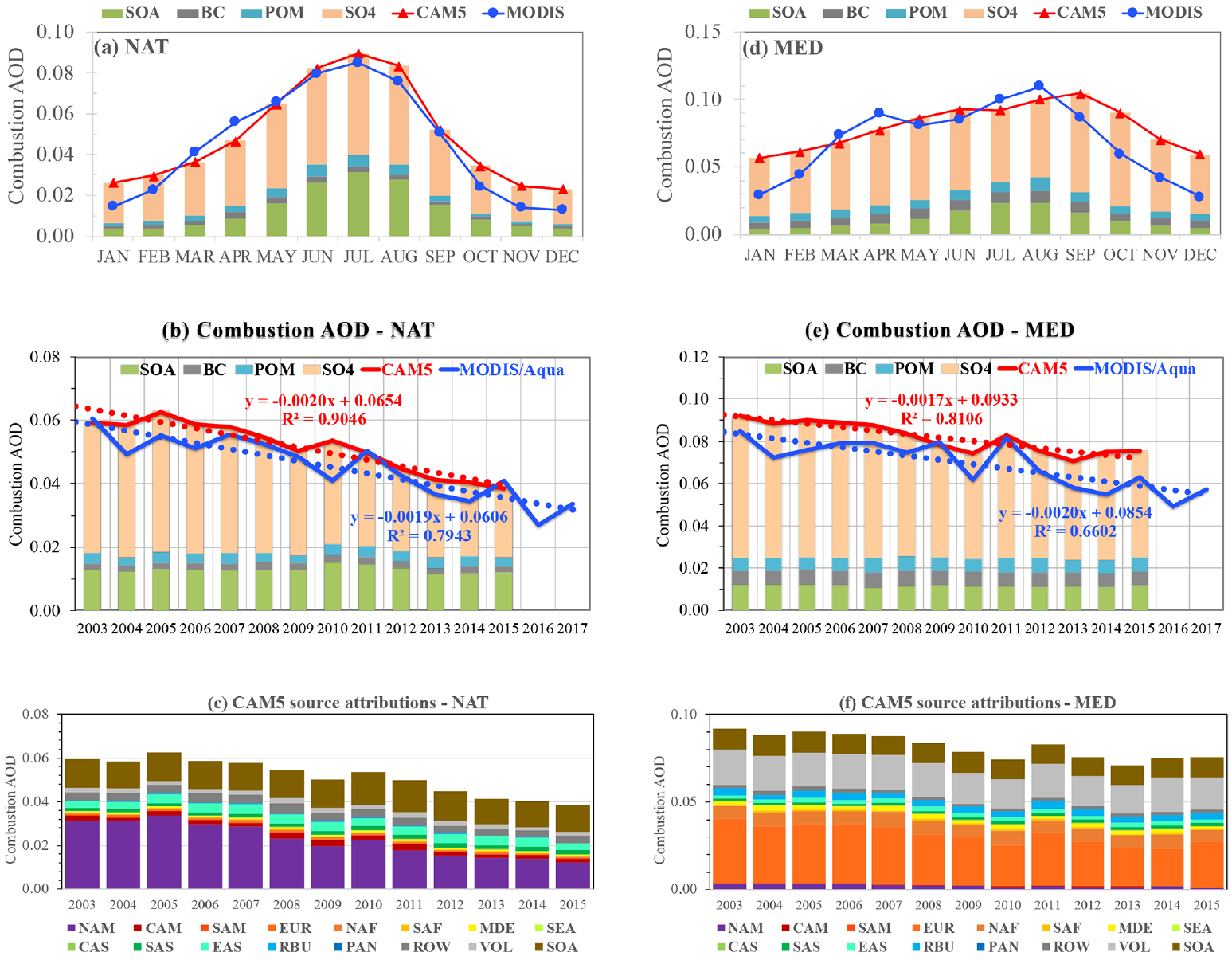
Climatological seasonal cycle of *τ*_c_ (**a, d**) interannual variability (solid line) and linear trend (dotted line) (**b, e**) retrieved by MODIS Aqua (blue) and simulated by the CAM5 model (red) in the North Atlantic Ocean (NAT, **a–c**) and the Mediterranean Sea (MED, **d–f**) outflow regions; the stacked bar shows CAM5 *τ*_c_ components of SO_4_ (excluding those produced from DMS chemistry), POM, BC, and SOA. Panels (**c, f**) show the source attributions of *τ*_c_ simulated by the CAM5 model.

Industrial and other anthropogenic emissions from North America (NAM) and Europe (EUR) have been declining steadily over the past several decades (see Fig. 2). Several studies have also consistently observed the decreasing trend of total AOD. Hsu et al. (2012) derived a decreasing trend of −0.028 and −0.027 per decade for *τ* over the eastern US and Europe, respectively, by using the SeaWiFS data over 1998–2010. On the east coast of North America, the MODIS Aqua retrievals show a decreasing trend of −0.028–−0.030 per decade (Alfaro-Contreras et al., 2017; Jongeward et al., 2016), whereas both the MODIS Terra and MISR data (after correcting the spurious increasing trend detected from the remote ocean regions) yield a decreasing trend of −0.022 per decade (Alfaro-Contreras et al., 2017). Over the Mediterranean Sea, Alfaro-Contreras et al. (2017) detected a decreasing trend of −0.025 per decade (MODIS Aqua and MISR) and −0.020 per decade (MODIS Terra). Figure 8 shows the MODIS Aqua retrievals and the CAM5 simulations of the climatological seasonal variations of *τ*_c_ (panels a, d), as well as the time series of annual mean *τ*_c_ (middle panels) in the North Atlantic Ocean (NAT, panels a–c) and Mediterranean Sea (MED, panels d–f) regions. In these panels, stacked bars are included to show the CAM5-simulated relative contributions by sulfate, POM, BC, and SOA to the total combustion AOD. The source contributions based on the CAM5 tagged-source simulations are shown in panels (c) and (f). In the NAT region, the peak influence of MODIS-retrieved *τ*_c_ occurs in July, which is about a factor of 4 larger than that in winter months. The CAM5 model captures the annual cycle of *τ*_c_ well with similar amplitude, although the modeled *τ*_c_ is generally higher than the MODIS retrieval in winter and fall months. The CAM5 model also reveals that the summer *τ*_c_ peak results mainly from increased sulfate and SOA, presumably due to more active photochemistry and higher relative humidity in summer. In the MED region, the peak of combustion AOD occurs in August for MODIS but September for CAM5. In winter and fall months, the CAM5 *τ*_c_ is higher than the MODIS retrieval, yielding a smaller amplitude of the CAM5 annual cycle. Similar to the NAT region, the summer *τ*_c_ peak in the MED region also results from increased sulfate and SOA. In both the NAT and MED regions, MODIS and CAM5 consistently show the decreasing trends of annual *τ*_c_ from 2003 onward at a level of −0.017–−0.020 per decade (*p<*0.01), which is somewhat smaller than those for total optical depth *τ* using similar satellite data (Alfaro-Contreras et al., 2017; Jongeward et al., 2016). The CAM5 source attribution simulations suggest that about 95 % of *τ*_c_ variability can be explained by man-made sulfate from a single source region, i.e., North America for NAT and Europe for MED. In the MED outflow region, the volcanic emission (VOL) constitutes the second largest contribution, which, however, has no clear trend over the period.

The decreasing trends of *τ*_c_ in the NAT and MED outflow regions also depend on season, as shown in Table 3. In the NAT region, the MODIS Aqua retrievals show a decreasing trend of −0.040 per decade (*p<*0.01) and −0.021 per decade (*p<*0.01) in JJA and MAM, respectively, which is much greater than that in DJF (−0.005 per decade) and SON (−0.010 per decade). On the other hand, the CAM5 simulations show a statistically significant trend (*p<*0.01) in all seasons; however, the decreasing trends of −0.023 and −0.033 per decade in MAM and JJA are a factor of 2–3 stronger than the decreasing trends of −0.011 and −0.012 per decade in DJF and SON. The finding of the stronger trend in JJA and MAM than in DJF and SON is consistent with that for total AOD from Jongeward et al. (2016). In the MED outflow region, the MODIS retrievals show statistically significant trends of −0.027 per decade (*p<*0.01) and −0.025 per decade (*p<*0.01) in MAM and JJA, but −0.020 per decade (*p<*0.05) in SON. There is no significant trend in DJF. In this region, the CAM5 simulations are consistent with the MODIS retrievals in terms of seasonal variability but with a smaller magnitude of the decreasing trend (e.g., −0.015 to −0.022 per decade or 20 %–30 % lower).

#### 3.3.2 Increasing trends of *τ*_c_ in the outflows of South Asia and the Middle East

**Figure 9.**
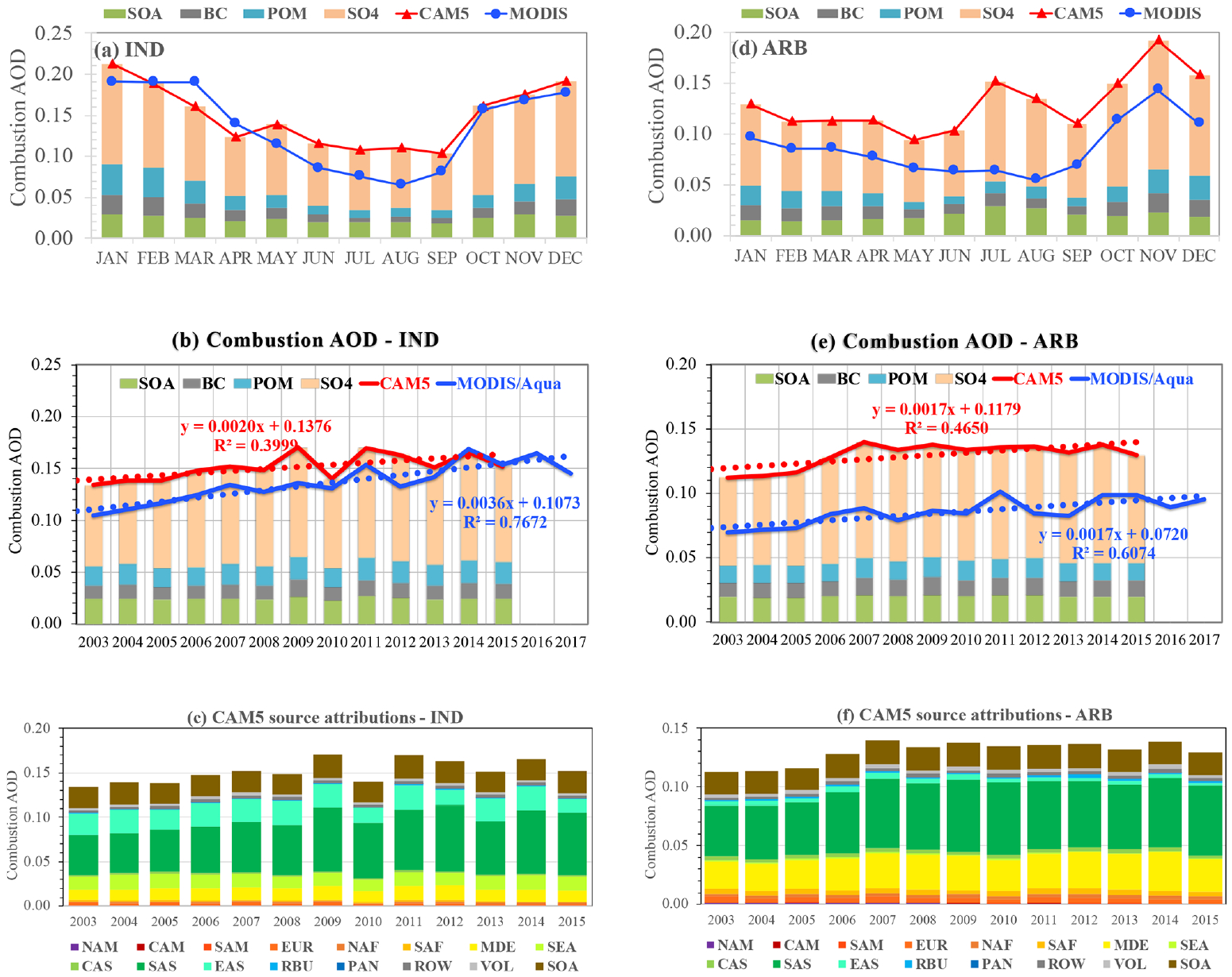
Same as Fig. 8 but for the tropical Indian Ocean–Bay of Bengal (IND, **a–c**) and the Arabian Sea (ARB, **d–f**) outflow regions.

In contrast to North America and Europe where combustion emissions have been decreasing over the past decades, combustion emissions in South Asia (SAS) and the Middle East (MDE) have increased relatively steadily (see Fig. 2). Li et al. (2017) show with satellite data that India may have sur-passed China in recent years by becoming the largest emitter of anthropogenic sulfur dioxide. Proestakis et al. (2018) detected from analyzing CALIOP data an increasing trend of +0.033 per decade for total AOD in South Asia from 2007 to 2015. SeaWiFS data suggest a strong increasing trend of +0.092 per decade on the Arabian Peninsula and +0.063 per decade in northern India for total AOD during 1998–2010 (Hsu et al., 2012). We show in Fig. 9 that the increase in combustion emissions in South Asia and the Middle East has caused an increasing *τ*_c_ trend over the tropical Indian Ocean and the Bay of Bengal (IND) as well as the Arabian Sea (ARB). In the IND region, both the MODIS retrievals and the CAM5 simulations show higher *τ*_c_ during October–March. Although the CAM5 *τ*_c_ is higher than the MODIS *τ*_c_ by about 0.035 or 35 % in 2003, the difference has reduced since then, becoming negligible in 2013–2015. Thus, the MODIS retrievals yield a much larger increasing trend of +0.036 per decade (*p<*0.01) for *τ*_c_ compared to the CAM5-simulated trend of +0.020 per decade (*p<*0.05). For comparison, Alfaro-Contreras et al., (2017) derived a total AOD trend of +0.031 *τ* per decade (MODIS Aqua), +0.050 *τ* per decade (MODIS Terra), and +0.022 *τ* per decade (MISR) over the Bay of Bengal. The CAM5 source attributions show that although South Asian sources are the largest contributor to the IND *τ*_c_, particularly in recent years, sources from other regions such as East Asia, Southeast Asia, and the Middle East also make non-negligible contributions. These four source regions combined (including SOA formation) explain 98.5 % of *τ*_c_ variability.

In the ARB region, the MODIS retrievals show a peak *τ*_c_ in October–December, while the CAM5 simulations show an additional second peak in July–August. The seasonal variation of combustion aerosol is mainly driven by sulfate, as suggested by the CAM5 simulations. The CAM5 *τ*_c_ is consistently higher than the MODIS retrieval throughout the year, and on an annual mean basis *τ*_c_ is higher by about +0.043 or 48 %. Despite this difference in the magnitude of *τ*_c_, an increasing trend with a similar magnitude of +0.017 per decade is derived from both the MODIS retrievals (*p<*0.01) and the CAM5 simulations (*p<*0.05). The CAM5 source attribution simulations further imply that the increasing trend in the ARB regions is largely driven by the increase in emissions from both South Asia and the Middle East. These two regions combined explain 94.4 % of *τ*_c_ variability. Our derived increasing *τ*_c_ trend of +0.017 per decade is a factor of 2–3 smaller than the +0.037 to +0.051 per decade for total AOD derived from previous work with MODIS (Terra and Aqua) and MISR retrievals (Alfaro-Contreras et al., 2017).

The trends in both regions show strong seasonal variations, as shown in Table 4. In the IND outflow region, both the MODIS retrievals and the CAM5 simulations suggest much stronger trends with higher statistical significance in the pre-monsoon seasons (MAM and DJF) than during SON. In the ARB region, on the other hand, the MODIS Aqua retrievals show a stronger increasing trend of +0.031 per decade in SON than the +0.023–0.025 per decade in DJF and MAM for *τ*_c_. In contrast, the CAM5 results show an increasing trend of +0.031 per decade (*p<*0.05) and +0.026 per decade (*p<*0.01) in MAM and DJF, respectively, but no significant trend in SON and JJA.

#### 3.3.3 Non-monotonic trends of *τ*_c_ in East Asian outflows

**Figure 10.**
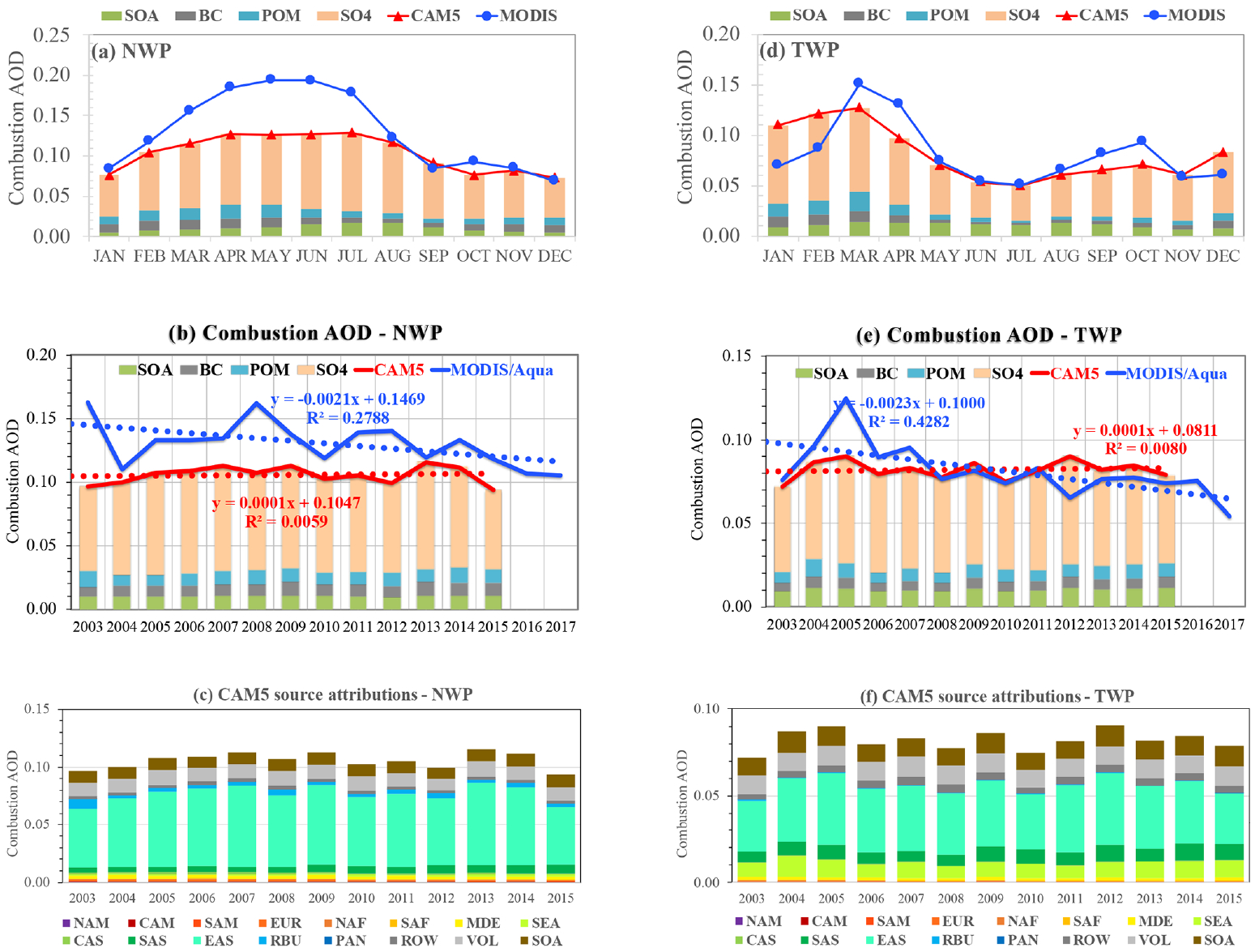
Same as Fig. 8 but for the northwestern Pacific Ocean (NWP, **a–c**) and the tropical western Pacific (TWP, **d–f**) outflow regions.

East Asia is the largest emitter of combustion aerosol and precursors in the world, as shown in Fig. 2. As discussed earlier, however, the regional emissions have followed a complicated trajectory in recent decades. De Leeuw et al. (2018) analyzed satellite AOD data over mainland China from 1995 to 2015 and found a distinct increase in AOD from 1995 to 2000 but a decreasing AOD since about 2011. Proestakis et al. (2018) found that total AOD over eastern China has decreased from 2007 to 2015 at a rate of −0.050 per decade based on CALIOP data. Observations from a large environmental monitoring network since 2013 have shown a general 30 %–50 % decrease in annual mean PM_2.5_ (particulate matter with diameter less than 2.5 μm) across China over the 2013–2018 period, which is consistent with concurrent observations of SO_2_ and carbon monoxide (CO) (Zhai et al., 2019). While the declining PM_2.5_ trend is mainly attributable to drastic controls on coal combustion, meteorology makes a secondary but still significant contribution to the declining trend of PM_2.5_ (Chen et al., 2018, 2019; Cheng et al., 2019; Zhai et al., 2019). The manner in which the outflows of East Asian emissions influence the northwestern Pacific Ocean (NWP) and tropical western Pacific Ocean (TWP) is shown in Fig. 10 from both the MODIS retrievals and CAM5 simulations. It reveals that the MODIS observed annual cycle in the NWP region has much higher *τ*_c_ in March–July than in other months. The MODIS *τ*_c_ is higher than the CAM5-modeled *τ*_c_ by 35 %–53 % during March–July, but the two datasets agree within 4 %–22 % in the other months. This results in a much weaker seasonal variation of *τ*_c_ in CAM5 than in MODIS. Anderson et al. (2005) compared MODIS FMF with in situ measurements of the small-mode fraction during the 2001 ACE-Asia campaign and found that the MODIS FMF is biased high by about 0.2. Although about 20 % of this high FMF bias for that specific year could be due to anomalous behavior in the MODIS Terra “side-B” electronics, the rest would be attributed to the assumption of a spherical shape for dust in the MODIS retrieval (Anderson et al., 2005). It is thus possible that the large MODIS–CAM5 difference during the dust season (March–May) could at least be partially attributed to the spherical dust assumption in the MODIS algorithm. On the other hand, the higher MODIS *τ*_c_ in June and July is likely a result of cloud contamination and limited sampling due to the more persistent presence of clouds. Over the period of 2003–2017, the MODIS retrievals show a decreasing trend of −0.021 per decade (*p<*0.05) in NWP. The MODIS retrievals also appear to show a stronger decreasing trend in the latter part of this period starting 2007. Our result is generally consistent with findings from other studies, in which the trajectory of AOD changed from increasing to decreasing at a certain pivot point. For example, Zhang et al. (2017) analyzed MODIS data over the northwestern Pacific Ocean and found that AOD has undergone a shift from an increasing trend over 2000–2007 to a decreasing trend over 2007–2015. Alfaro-Contreras et al. (2017) analyzed several satellite retrievals of AOD for total aerosol and found no consistent trend in coastal China (−0.035, +0. 001, −0.010 per decade for MODIS Aqua, MODIS Terra, and MISR, respectively). Sogacheva et al. (2018) combined AOD data from the Along-Track Scanning Radiometer (ATSR) and MODIS and found that AOD across China increased significantly from 1995 to 2006 but decreased gradually between 2011 and 2017, which reflects the increased emissions due to rapid economic development and the decreased emissions due to effective emission control regulations. For comparison, the CAM5 simulations show no statistically significant trend. The CAM5 simulations also suggest that 95 % of *τ*_c_ variability can be explained by a single source region in East Asia.

In the TWP region, although the MODIS retrievals and CAM5 results agree well from May to December, they differ in January to April, the months of peak *τ*_c_. The MODIS retrievals show peaks in March–April, whereas the CAM5 simulation suggests peaks during January–March. Over the period of 2003–2017, the MODIS retrievals show a decreasing trend of −0.023 per decade (*p<*0.01) in TWP. Note that the MODIS *τ*_c_ in 2005 was higher by 31 %–92 % than that in any other year. Previous studies (Yuan et al., 2011; Yu et al., 2008) have traced the 2005 high aerosol loading to volcanic activity east of the Philippines. Clearly, the CAM5 model underestimates this 2005 *τ*_c_ peak by 28 %, although the model produces the magnitude of MODIS *τ*_c_ in the other years well. This may suggest that the CAM5 model underestimated the 2005 volcanic emissions in this region. Compared to the MODIS retrievals, the CAM5 simulations show no statistically significant trend of combustion AOD. Similar to the NWP region, 95 % of *τ*_c_ variability can be explained by emissions from East Asia. Boreddy et al. (2018) examine the 2001–2012 data record of surface carbonaceous aerosols measured in Chichijima, a remote island (27°04′ N, 142°13′ E) just near the northeastern corner of our TWP box. Their data show that carbonaceous aerosol has maxima in winter to spring and minima in summer, which is consistent with the CAM5 model simulation. They also detected statistically significant increasing trends of OC and water-soluble OC, probably due to the enhanced photochemical oxidation of biomass burning and biogenic VOCs during long-range transport, but a decreasing trend of elemental carbon (EC) probably associated with the decrease in fossil fuel sources. For comparison, the CAM5 simulations over the same period show weakly increasing but not statistically significant trends for BC, POM, and SOA. The above analyses suggest that the model would need to improve its simulations of combustion aerosol trends in this region.

#### 3.3.4 Interannual variability in smoke- or dust-dominated outflows

**Figure 11.**
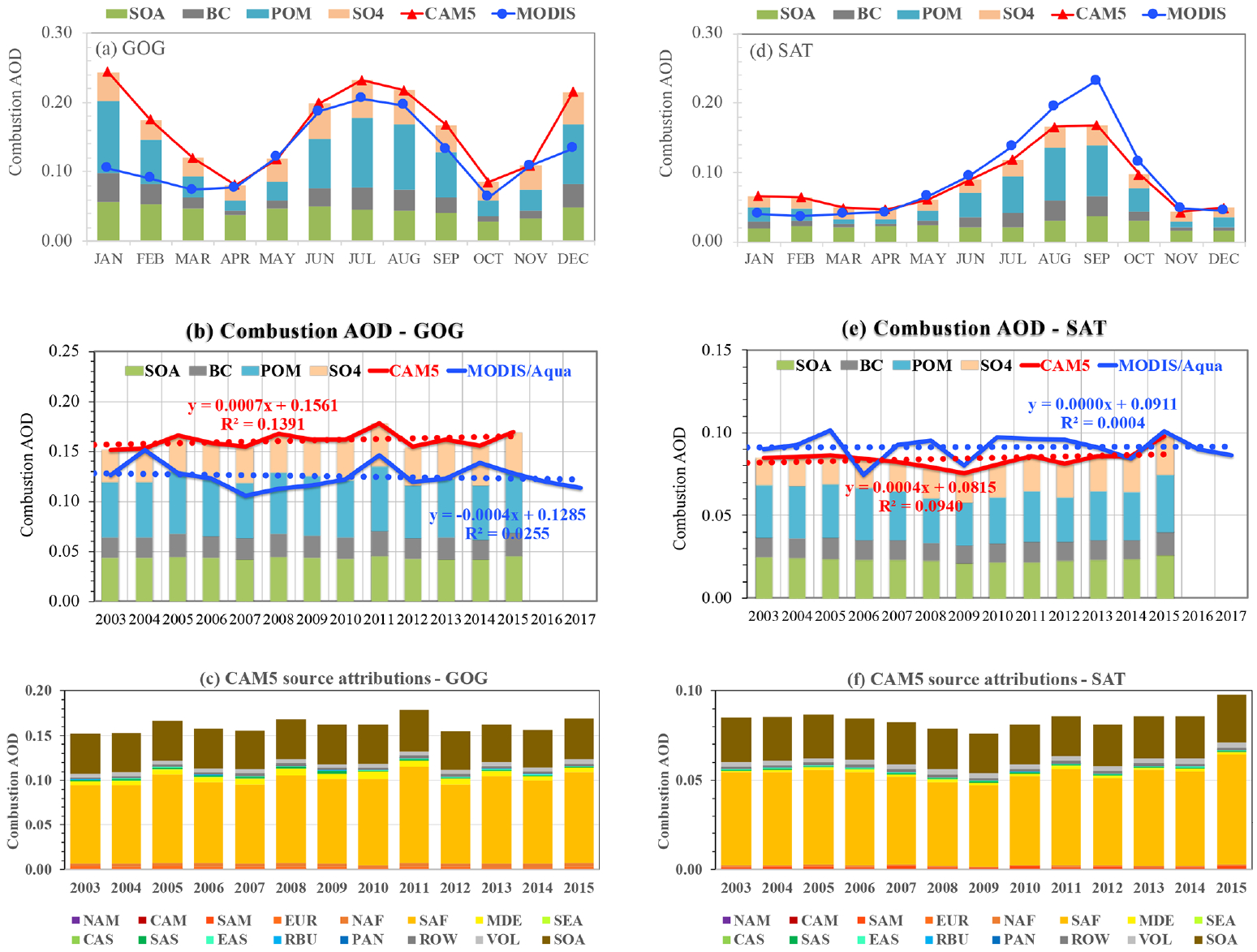
Same as Fig. 8 but for the Gulf of Guinea (GOG, **a–c**) and the southeastern Atlantic Ocean (SAT, **d–f**) outflow regions strongly influenced by biomass burning smoke.

In comparison to industrial emissions, biomass burning emissions often show large fluctuations from year to year but without a steady trend because of their strong dependence on meteorological conditions. Figure 11 shows results from our analysis of the MODIS retrievals and CAM5 simulations in two outflow regions where biomass burning smoke from southern Africa (SAF) contributes significantly to *τ*_c_, namely the Gulf of Guinea (GOG) and the southeastern Atlantic Ocean (SAT). In the GOG outflow, the seasonal variation of combustion aerosol shows a bimodal distribution (Fig. 11a), with a peak in December–January and another peak in June–August attributed to northern equatorial Africa and southern equatorial Africa, respectively. Although CAM5 and MODIS agree within 0 %–11 % during the April–November period, CAM5 combustion AOD is 60 %–130 % higher than the MODIS-derived combustion AOD from December to March. In the SAT outflow region, combustion AOD peaks in August–September as shown by both MODIS and CAM5, although MODIS is 18 %–38 % higher than the CAM5 simulation (Fig. 11d). In both regions, the MODIS retrievals and CAM5 simulations show interannual variations but no trends (Fig. 11b and e). The CAM5 source attribution simulations (Fig. 11c and f) suggest that emissions from southern Africa (SAF) can explain more than 94 % and 97 % of *τ*_c_ variability in the GOG and SAT outflow region, respectively. Although the CAM5 *τ*_c_ is on average lower than the MODIS retrieval by only 0.008 or 9 % in the SAT outflow region, the CAM5 *τ*_c_ is 0.035 or 28 % higher than the MODIS retrieval in the GOG outflow region. It is important to note that GOG and SAT are affected by biomass burning from northern and southern equatorial Africa, respectively. However, northern and southern equatorial Africa has been aggregated together as a single broad source region (SAF) in this study. It is possible that the biomass burning emission in southern equatorial Africa is reasonably captured by CAM5, whereas the emission in northern equatorial Africa may be overestimated by the model. On the other hand, the smaller MODIS *τ*_c_ in GOG during the December–March period might also allude to another possibility that our MODIS-based dust–combustion separation algorithm may have attributed part of combustion aerosol to dust (Yu et al., 2009, 2019).

**Figure 12.**
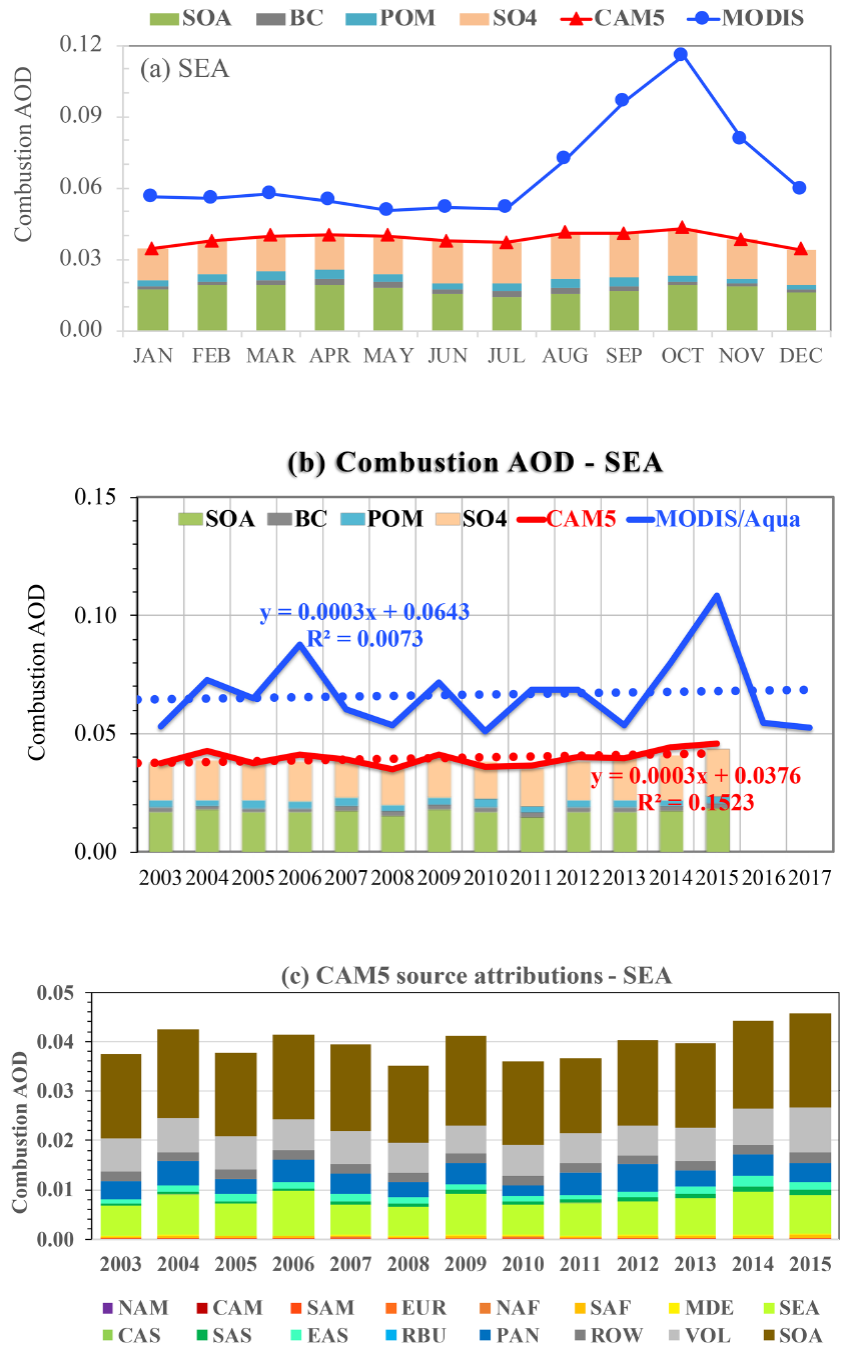
Same as Fig. 8 but for the southeastern Asia (SEA, **d–f**) outflow regions strongly influenced by biomass burning smoke.

In the SEA outflow region, the MODIS combustion AOD is consistently higher than the CAM5 simulation throughout a year (Fig. 12a). Although the MODIS retrievals show a strong influence of biomass burning smoke during the August–November period with a peak in October, the CAM5 simulation shows a fairly flat combustion AOD throughout a year. This suggests that the CAM5 model substantially underestimates smoke emissions in southeastern Asia. As shown in Fig. 12b, the MODIS retrievals show very large interannual variability, with elevated *τ*_c_ values corresponding to El Niño years (e.g., 2004, 2006, 2009, and 2015) when drought conditions associated with El Niño drove up biomass burning events in Indonesia (Field et al., 2016; Pan et al., 2018). The years 2015 and 2006 had higher *τ*_c_ than the years 2009 and 2004, which cannot be explained by the El Niño index alone, but instead is believed to result from interactions of El Niño and the Indian Ocean Dipole (IOD) that determine atmospheric circulations and rainfall (Pan et al., 2018). Specifically, 2015 and 2006 were years that IOD was in phase with El Niño (so-called eastern Pacific type of El Niño), whereas 2009 and 2004 were years that IOD was out of phase or weakly in phase with El Niño (so-called central Pacific type of El Niño) (Pan et al., 2018). In comparison, the CAM5 simulations are on average 42 % lower than the MODIS retrievals and show much smaller interannual variability. Carbonaceous aerosol (POM, BC, and SOA) accounts for about half of *τ*_c_ in the region. The source attribution analysis of the CAM5 simulations (Fig. 12c) shows that sources from Southeast Asia and SOA, combined, explain about 88 % of the *τ*_c_ variability. The substantially lower magnitude and smaller seasonal and interannual variability of *τ*_c_ by CAM5 suggest that the fire emissions in Southeast Asia are likely to be underestimated in the model. In particular, the CAM5 POM emission from Southeast Asia in 2015 was even 25 % lower than that in 2014, which is in stark contrast to the MODIS observations. It is also important to note that the POM-to-SOA ratio in the southeastern Asia is substantially smaller than that in the Gulf of Guinea and southeastern Atlantic Ocean.

**Figure 13.**
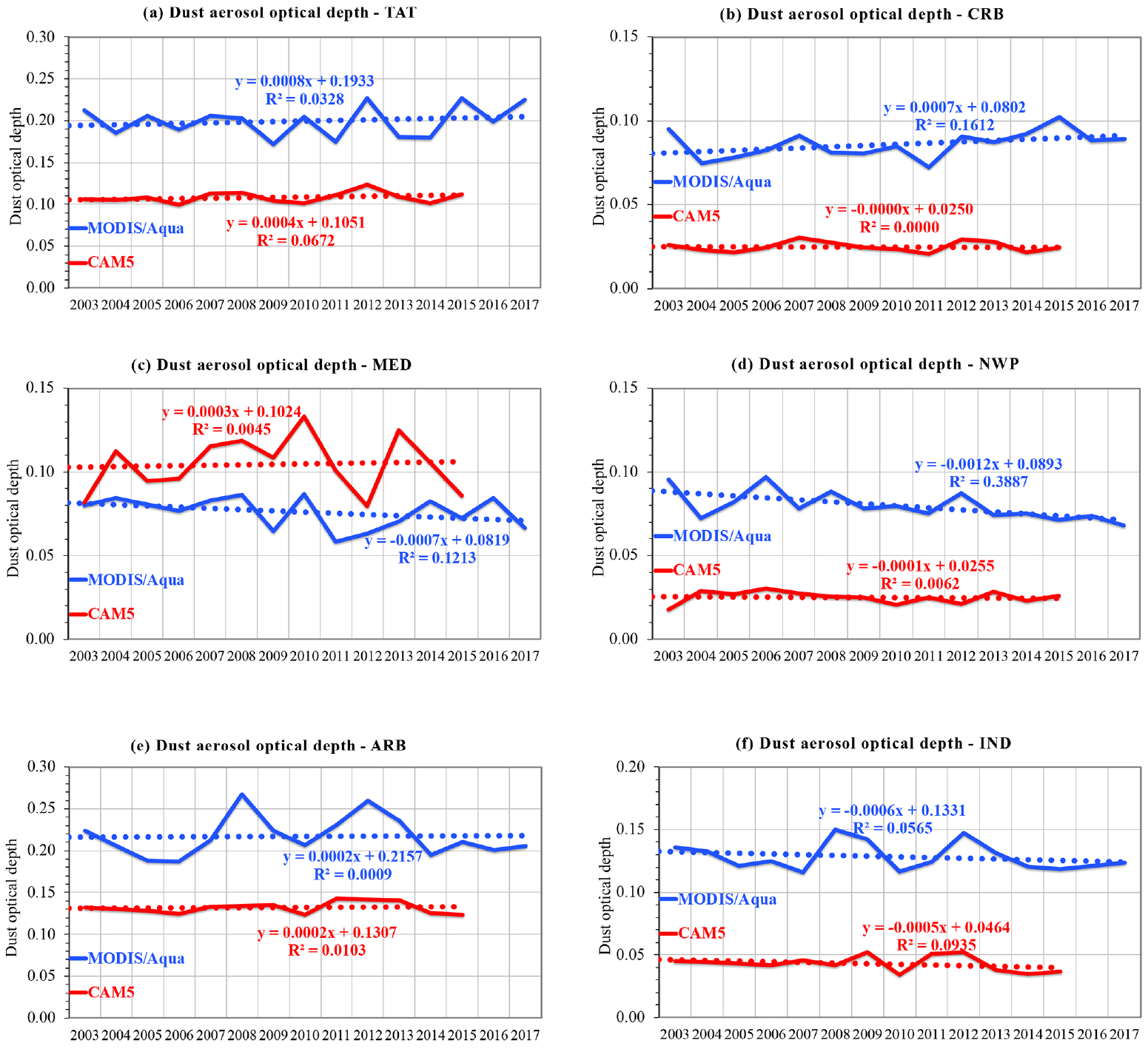
Interannual variability of *τ*_d_ in six major outflow regions, as revealed by the MODIS Aqua retrievals (blue lines) and CAM5 simulations (red lines): (**a**) the tropical Atlantic Ocean – TAT, (**b**) the Caribbean Basin – CRB, (**c**) the Mediterranean Sea – MED, (**d**) the northwest Pacific Ocean – NWP, (**e**) the Arabian Sea – ARB, and (**f**) the tropical Indian Ocean and the Bay of Bengal – IND.

Similar to the biomass burning smoke outflow regions discussed above, *τ*_d_ in six major dust outflow regions also displays large interannual variability, but generally no clear trend, based on both the MODIS retrievals and CAM5 simulations, as shown in Fig. 13. One exception is the northwestern Pacific (NWP) outflow region influenced mainly by Asian dust emissions where MODIS *τ*_d_ shows a decreasing trend of −0.012 per decade or −1.5 % yr^−1^ (*p<*0.05). Further analysis shows that this decreasing trend of annual *τ*_d_ results from a decreasing trend of −0.034 per decade or −2.6 % yr^−1^ (*p<*0.05) in MAM but negligible trends in the other seasons. These decreasing trends of dust are smaller in magnitude than that detected from the Asian Dust Network (AD-Net) lidar observations over Japan, i.e., −4.3 % yr^−1^ and 2.5 % yr^−1^ for MAM and the annual mean, respectively (Shimizu et al., 2017). Our results are also consistent with trends of dust emissions over East Asia and China in particular as documented in the literature. For example, Song et al. (2016) showed that the spring dust storm frequency in arid and semiarid regions of China has decreased by 15.45 storms per year on average over the period of 1982 to 2007. Fan et al. (2014) showed that the decrease in springtime dust storms in Inner Mongolia, northern China, from 1982 to 2008 was correlated with advanced vegetation growth, with 1 d earlier green-up data corresponding to a 3 % decrease in spring dust storms. An et al. (2018) analyzed the frequency of sand and dust storms observed from ground stations in East Asia during 2007–2016 and found a decreasing trend that is associated with an increase in vegetation coverage and the weakening of the polar vortex. Proestakis et al. (2018) detected from analyzing CALIOP data a stronger decreasing trend of −0.021 per decade for annual average *τ*_d_ during the period of 2007–2015 in East China (a domain including both land and the adjacent eastern China Sea). Although Proestakis et al. (2018) also found a decreasing trend of −0.015 per decade over South Asia (a domain including the India subcontinent, the Bay of Bengal, and the northern Indian Ocean), the MODIS retrievals in our study show no statistically significant trend in the IND outflow region. Finally, although CAM5 *τ*_d_ in the MED outflow region is 36 % higher than the MODIS retrieval, it is a factor of 2–3 smaller than the MODIS retrieval in other dust outflow regions. This low bias of CAM5 *τ*_d_ in the regional analysis is expected from the global plots of Fig. 4.

## 4 Conclusions and discussion

We have reassessed the MODIS FMF-based algorithm that partitions total AOD over ocean into combustion aerosol, dust, and marine aerosol by using the Collection 6 data from both Terra and Aqua. By using the derived C6-specific characteristic FMF for the individual aerosol types, we produced the MODIS Terra and MODIS Aqua retrievals of over-ocean *τ*_c_ and *τ*_d_ over a period of 15+ years. The MODIS retrievals were compared with the CAM5 simulations. We then analyzed the MODIS Aqua retrievals from 2003 to 2017 to examine the interannual variability and possible trends of *τ*_c_ and *τ*_d_ over 13 major continental outflow regions around the globe. This MODIS-based analysis was complemented by the CAM5 source-tagging simulations from 2003 to 2015 to identify the source attributions. Major results derived from this study include the following.

1. The characteristic FMF for combustion aerosol, dust, and marine aerosol is derived to be 0.89, 0.31, and 0.48, respectively, from the MODIS Aqua C6 product. Corresponding values derived from the MODIS Terra C6 product are 0.92, 0.26, and 0.55, respectively. Significant changes in the characteristic FMF values for dust and marine aerosol have occurred throughout the evolution from C4 and C5 to the latest C6. Differences in instrument calibrations and changes in the details of the aerosol retrievals may have created these differences between Aqua and Terra, as well as between different data collections. The bottom line is that the MODIS data in each collection should be used in a consistent way to avoid introducing unwanted errors. Applying the MODIS-based characteristic FMF directly to VIIRS observations is also not encouraged.
2. The CAM5 combustion AOD *τ*_c_ simulations show a close agreement with the MODIS retrievals over ocean, with the global ocean average being ~13 % lower than the MODIS *τ*_c_. However, the CAM5 simulations of dust AOD *τ*_d_ over ocean account for no more than ~38 % of the MODIS retrievals even when possible cloud contamination in the MODIS retrievals is empirically accounted for.
3. In contrast to the MODIS Aqua retrievals, the MODIS Terra retrievals show a statistically significant, increasing trend of combustion AOD in the remote ocean regions and global ocean average. This trend is considered to be spurious, possibly due to the imperfect instrument calibrations of the MODIS Terra instrument and other issues, as presented and discussed in Levy et al. (2018). It is thus recommended that the MODIS Aqua retrievals only be used to examine regional AOD trends.
4. The MODIS retrievals and CAM5 simulations consistently yield a decreasing trend of −0.017 to −0.020 per decade for the combustion AOD (*p<*0.01) over the North Atlantic Ocean (NAT) and the Mediterranean Sea (MED) that is respectively influenced by the reduction of combustion-related emissions from North America and Europe. In these regions, MODIS and CAM5 are also consistent in depicting the seasonal variation of the trend.
5. In contrast, both MODIS retrievals and the CAM5 simulations display increasing *τ*_c_ trends over the tropical Indian Ocean–Bay of Bengal (IND) and the Arabian Sea (ARB) due to the influence of increased anthropogenic emissions from South Asia and to a lesser degree from the Middle East in recent years. The MODIS-based trend of +0.036 per decade (*p<*0.01) in the IND outflow region is nearly 2 times the CAM5-derived trend (*p<*0.05). Although the CAM5 *τ*_c_ is higher than the MODIS retrieval by about 48 % over the ARB outflow, the two datasets give a similar increasing trend of +0.017 per decade (*p<*0.01).
6. The MODIS retrievals show a decreasing trend of −0.021 per decade (*p<*0.05) and −0.023 per decade (*p<*0.01) for combustion AOD in the northwestern Pacific Ocean (NWP) and tropical western Pacific Ocean (TWP), respectively, both being influenced by anthropogenic emissions from East Asia. The decreasing trend appears to be stronger after 2008 (NWP) and 2005 (TWP), consistent with the transition from an increase to a decrease in SO_2_ emissions from China as documented in previous studies. The MODIS retrievals also show a decreasing trend of −0.012 per decade (*p<*0.05) for dust AOD in the NWP outflow region, which is consistent with the detected decreasing trends of dust emissions and storm frequency over China. However, the CAM5 simulations do not reproduce these declining trends.
7. In other outflow regions strongly influenced by biomass burning smoke or dust, neither MODIS retrievals nor CAM5 simulations show statistically significant trends, arguably due to the episodic nature of smoke and dust emissions. The MODIS-observed interannual variability is generally larger than the CAM5 simulations. It is also found that the CAM5–MODIS difference in aerosol optical depth is in general significantly larger in smoke and dust outflow regions than those affected mainly by fossil fuel emissions, highlighting the difficulty in quantifying episodic dust and smoke emissions and their evolution.

The aerosol trend detection is restricted by several issues associated with satellite remote sensing, such as instrument calibration, limited sampling obscured by the presence of clouds and sun glint, and various assumptions in the retrieval algorithms (Li et al., 2009). To minimize the influences of these limitations on the trend analysis, we have used the MODIS Aqua data products that are derived from consistent retrieval algorithms with robust calibrations. Long-term data records are required to aggregate sufficient sampling (Chedin et al., 2018), with the required length of data record depending on the region. In downwind outflow regions of North American and western European combustion sources, the downward trends are statistically significant because of continuous emission reductions. The upward trends over the tropical Indian Ocean and Arabian Sea are also statistically significant due to the continuous increase in combustion emissions. For these regions the length of the data record might not be very critical, and the 15 years of data used in this study are deemed to be adequate. However, in regions where emissions have undergone non-monotonous changes or the emissions trends are entangled with meteorological variability in a complex way, the 15 years of data are not adequate. We showed that MODIS observations over the western Pacific Ocean display a less statistically significant decreasing trend of combustion aerosol than that over the tropical Indian Ocean and Arabian Sea because the emissions in East Asia, particularly in China, have increased and then decreased over the last 2 decades as a result of economic development and implementation of air pollution control regulations. We also did not detect statistically significant trends for the majority of dust and smoke outflow regions, arguably because spo-radic emissions of dust and smoke and their transport are strongly modulated by meteorological conditions. Although Chedin et al. (2018) suggest that 14–15 years of the IASI data record would be adequate to examine the dust trend over Africa, the use of a 15-year MODIS dataset in this study did not yield a trend of dust near the coast of North Africa. This discrepancy may have resulted from inherent differences in satellite sampling and instrument sensitivity, among others. Clearly, a longer MODIS data record is required to reliably detect any trends of smoke and dust aerosol.

Our analysis shows that the interannual variability of combustion AOD in major outflow regions is largely consistent with that of emissions from upwind continental sources. Although this finding highlights the importance of correctly characterizing the variability of combustion emissions, the role of meteorology in transporting aerosol from combustion sources downwind to outflow regions cannot be ruled out. For dust and smoke aerosol, both their emissions and transport are strongly modulated by meteorology and hence their variability and trends must account for meteorological factors. Well-designed model sensitivity experiments are needed to disentangle meteorological variability from that of emissions.

In this study, we have categorized satellite observed total aerosol into three broad types, namely marine aerosol, combustion aerosol, and dust. We have shown that in comparison to an analysis of total AOD, this broad categorization has provided additional insights into the variability and trends of aerosol in terms of their sources, which is useful for guiding model improvement. Given the complexity of aerosol sources and transformation processes, such a broad aerosol categorization is still not adequate for fully understanding aerosol trends and variability. For example, “combustion” aerosol has been loosely defined in this study because it does not separate aerosol derived from fossil fuel and biomass burning or sulfate derived from industrial and volcanic activities. Although some effort is emerging in attempting retrievals of aerosol chemical composition from satellites (Li et al., 2019), an accurate and detailed characterization of chemical composition will most likely rely on an accumulation of in situ observations from suborbital platforms.

## Supplementary Material

Supplementary Material

## Figures and Tables

**Table 1.** Characteristic fine-mode fraction for individual aerosol types (*f*_c_, *f*_d_, and *f*_m_) as derived from the MODIS C6 data and GOCART simulations.

Aerosol type	MODIS Terra	MODIS Aqua	GOCART
Combustion (*f*_c_)	0.92	0.89	0.95
Dust (*f*_d_)	0.26	0.31	0.25
Marine (*f*_m_)	0.55	0.48	0.78

**Table 2.** Comparison of characteristic FMF for individual aerosol types as derived from different MODIS data collections (C6, C5, and C4). Values corresponding to C5 and C4 were taken from Yu et al. (2009).

Aerosol type	C6	C5	C4
Combustion (*f*_c_)	0.92	0.90	0.92
Dust (*f*_d_)	0.26	0.37	0.51
Marine (*f*_m_)	0.55	0.45	0.32

**Table 3.** Trends of combustion AOD (decade^−1^) with seasonal distinction derived from the MODIS Aqua retrievals and CAM5 simulations in NAT and MED outflow regions. A trend with a significance level of *p<*0.05 and *p<*0.01 is marked with * and **, respectively. A trend without an asterisk is considered not statistically significant.

Outflow region	NAT		MED	
	MODIS	CAM5	MODIS	CAM5
DJF	−0.005**	−0.011**	−0.007	−0.012
MAM	−0.021**	−0.023**	−0.027**	−0.022**
JJA	−0.040**	−0.033**	−0.025**	−0.018**
SON	−0.010*	−0.012**	−0.020*	−0.015**
Annual	−0.019**	−0.020**	−0.020**	−0.017**

**Table 4.** Similar to Table 3 but for trends of combustion AOD (decade^−1^) in IND and ARB outflow regions. A trend with a significance level of *p<*0.05 and *p<*0.01 is marked with * and **, respectively. A trend without an asterisk is considered not statistically significant.

Outflow region	IND		ARB	
	MODIS	CAM5	MODIS	CAM5
DJF	+0.050**	+0.047**	+0.025**	+0.031*
MAM	+0.062**	+0.028*	+0.023**	+0.026**
JJA	+0.003	−0.002	−0.009	−0.001
SON	+0.034*	+0.013	+0.031**	+0.014
Annual	+0.036**	+0.020*	+0.017**	+0.017**
