# Supplementary material for: Interannual variability and trends of combustion aerosol and dust in major continental outflows revealed by MODIS retrievals and CAM5 simulations during 2003–2017
Source: Atmos Chem Phys. Author manuscript; Available in PMC 2020 Nov 16. (PMC7668156; doi:10.5194/acp-20-139-2020)
Supplement: Supplementary Material [file NIHMS1643830-supplement-Supplementary_Material.pdf]

Supplement of Atmos. Chem. Phys., 20, 139–161, 2020
https://doi.org/10.5194/acp-20-139-2020-supplement
© Author(s) 2020. This work is distributed under
the Creative Commons Attribution 4.0 License.

Atmospheric Chemistry and Physics
Open Access

Supplement of

Interannual variability and trends of combustion aerosol and dust in major continental outflows revealed by MODIS retrievals and CAM5 simulations during 2003–2017

Hongbin Yu et al.

Correspondence to: Hongbin Yu (hongbin.yu@nasa.gov)

The copyright of individual parts of the supplement might differ from the CC BY 4.0 License.

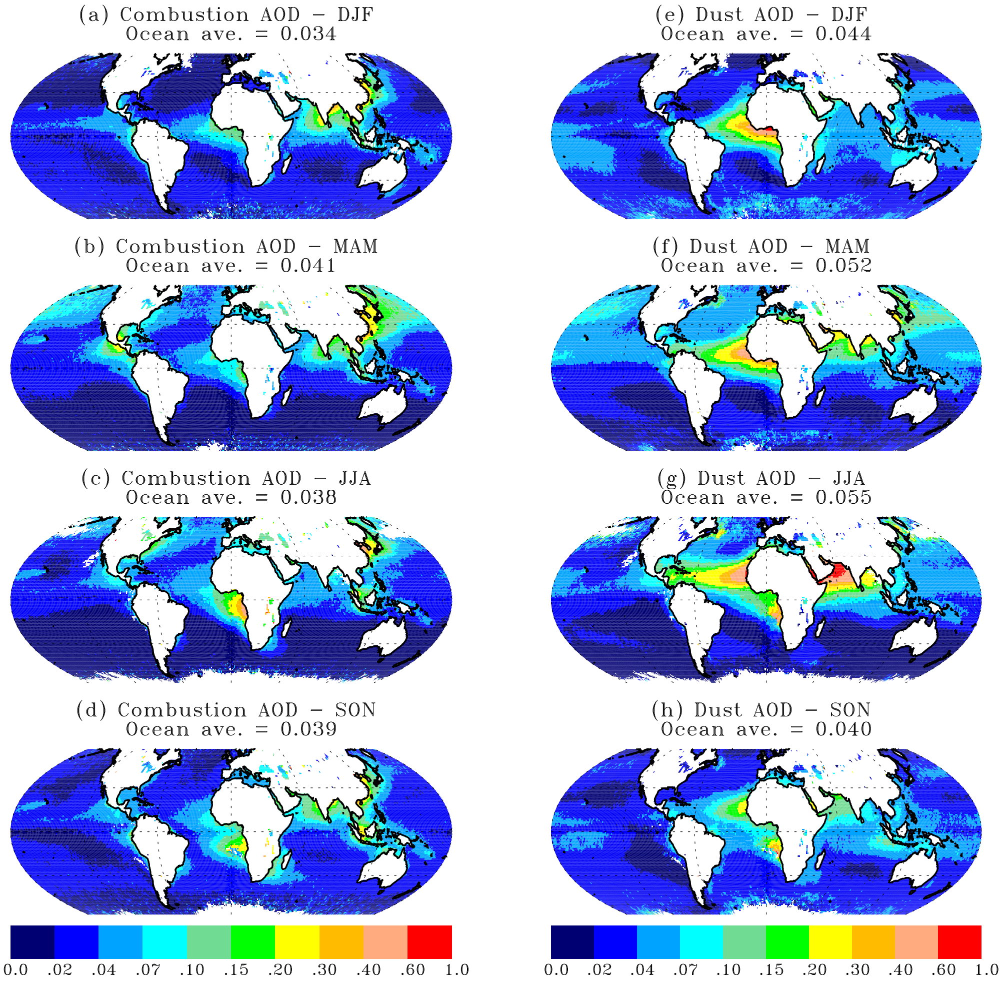

Figure S1: The MODIS/Aqua seasonal mean combustion AOD (τ_c, a - d) and dust AOD (τ_d, e - h) averaged over 2003-2015.

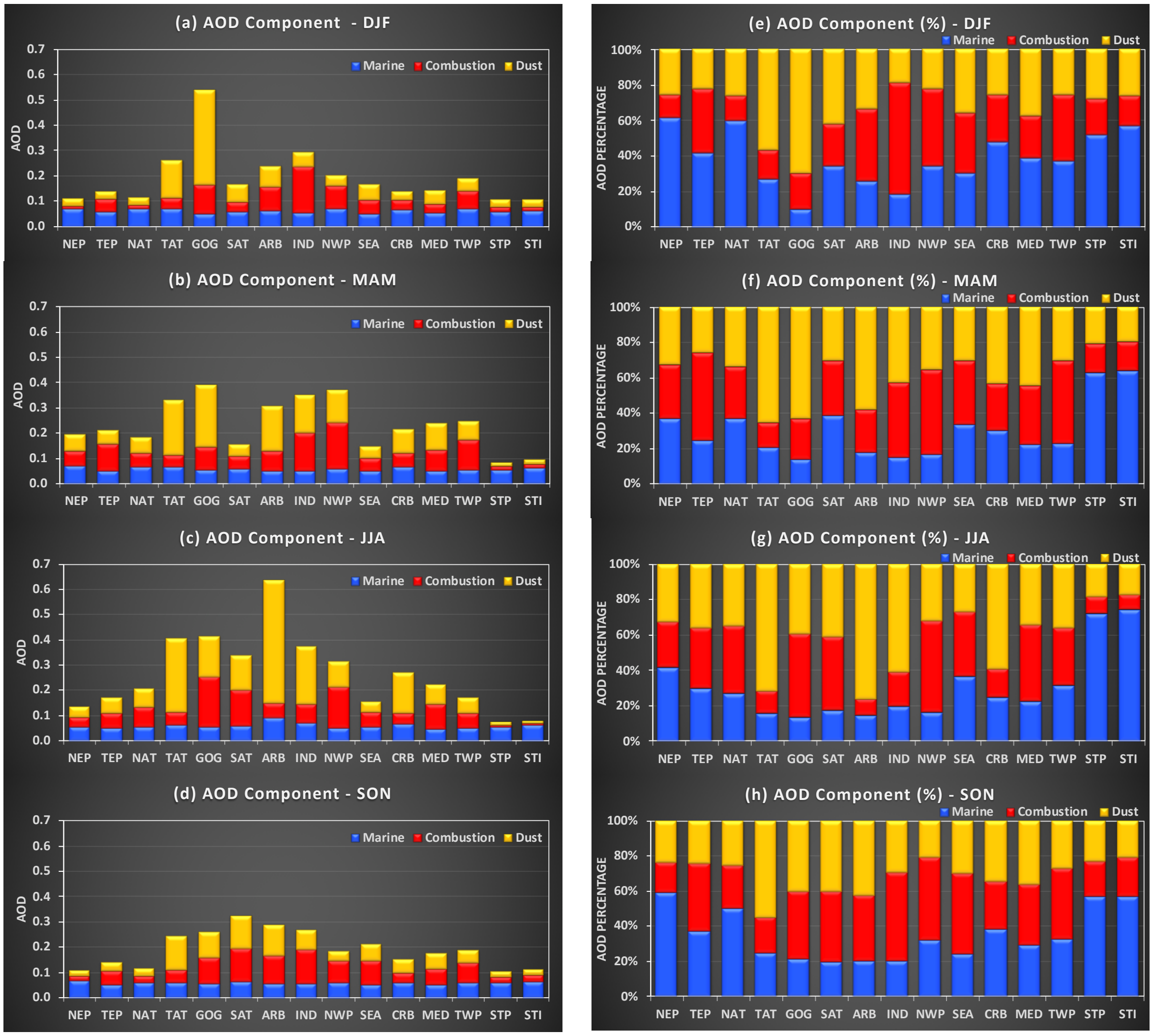

Figure S2: Aerosol component climatology with seasonal distinctions in the 15 regions as derived from 2003-2017 MODIS Aqua observations: (a - d) for magnitude of component AOD, and (e - g) for percentage of component AOD.

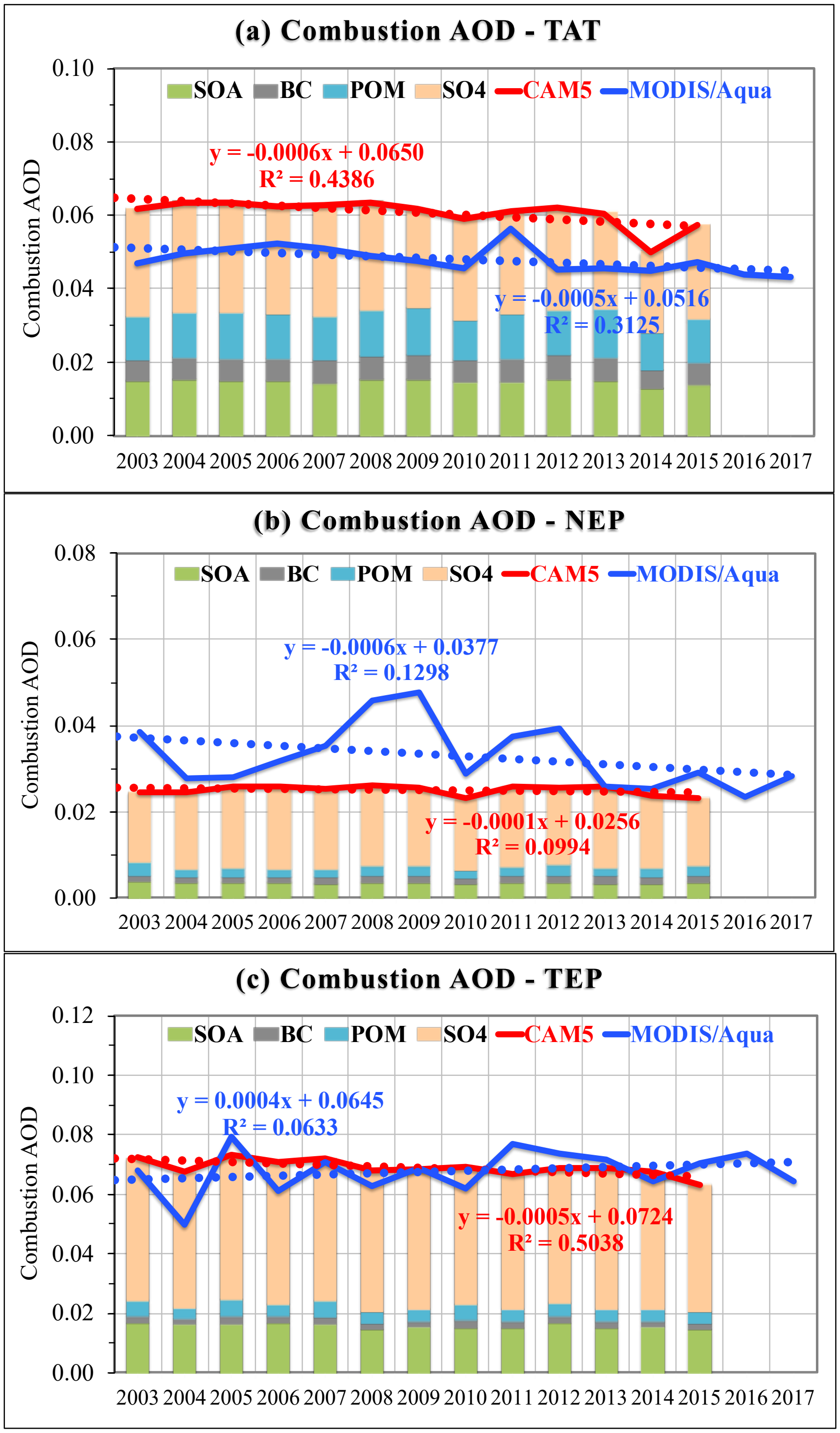

Figure S3: Interannual variability of combustion aerosol optical depth in (a) TAT, (b) NEP, and (c) TEP outflows derived from MODIS/Aqua and CAM5.

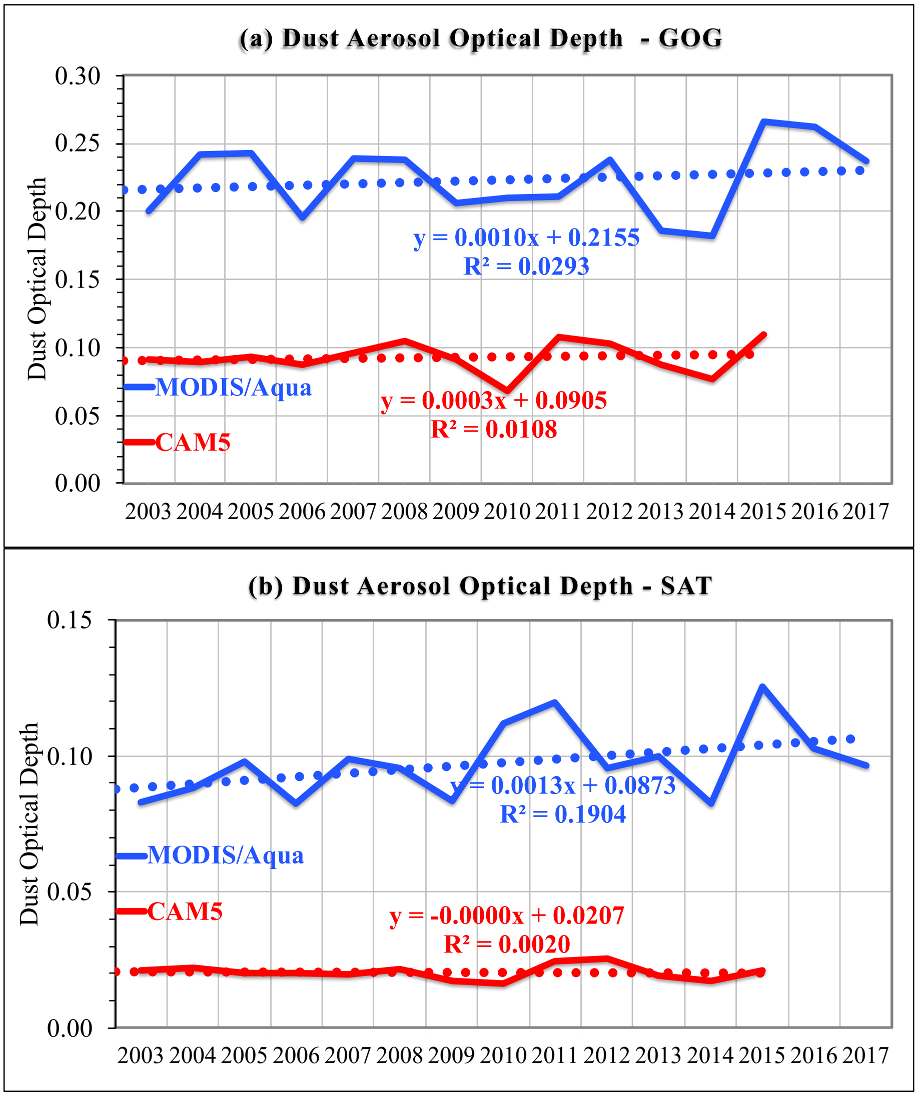

Figure S4: Interannual variability of dust aerosol optical depth in (a) GOG and (b) SAT outflows derived from MODIS/Aqua and CAM5.
